# FGF23 promotes renal calcium reabsorption through the TRPV5 channel

**DOI:** 10.1002/embj.201284188

**Published:** 2014-01-17

**Authors:** Olena Andrukhova, Alina Smorodchenko, Monika Egerbacher, Carmen Streicher, Ute Zeitz, Regina Goetz, Victoria Shalhoub, Moosa Mohammadi, Elena E Pohl, Beate Lanske, Reinhold G Erben

**Affiliations:** 1 University of Veterinary Medicine ViennaVienna, Austria; 2 New York University School of MedicineNew York, NY, USA; 3 Amgen Inc.Thousand Oaks, CA, USA; 4 Harvard School of Dental MedicineBoston, MA, USA

**Keywords:** calcium homeostasis, fibroblast growth factor-23, Klotho, renal calcium reabsorption, transient receptor potential vanilloid-5

## Abstract

αKlotho is thought to activate the epithelial calcium channel Transient Receptor Potential Vanilloid-5 (TRPV5) in distal renal tubules through its putative glucuronidase/sialidase activity, thereby preventing renal calcium loss. However, αKlotho also functions as the obligatory co-receptor for fibroblast growth factor-23 (FGF23), a bone-derived phosphaturic hormone. Here, we show that renal calcium reabsorption and renal membrane abundance of TRPV5 are reduced in Fgf23 knockout mice, similar to what is seen in αKlotho knockout mice. We further demonstrate that αKlotho neither co-localizes with TRPV5 nor is regulated by FGF23. Rather, apical membrane abundance of TRPV5 in renal distal tubules and thus renal calcium reabsorption are regulated by FGF23, which binds the FGF receptor-αKlotho complex and activates a signaling cascade involving ERK1/2, SGK1, and WNK4. Our data thereby identify FGF23, not αKlotho, as a calcium-conserving hormone in the kidney.

## Introduction

αKlotho (Klotho) is a single pass transmembrane protein, which shares sequence homology with family I β-glycosidases, including β-glucuronidases (Kuro-o *et al*, 1997). However, Klotho lacks essential active site glutamic acid residues typical for this family of glycosidases (Tohyama *et al*, 2004). The extracellular domain of Klotho, which consists of two type I β-glycosidase domains (KL1 and KL2), can be shed from the cell surface by membrane-anchored proteolytic enzymes, and released into the blood circulation (Imura *et al*, 2007). In addition, a soluble Klotho isoform can be produced by alternative splicing of the Klotho mRNA (Matsumura *et al*, 1998; Shiraki-Iida *et al*, 1998). This truncated *Klotho* gene product lacks exons 4 and 5 in mice (Shiraki-Iida *et al*, 1998). Klotho is mainly expressed in renal distal convoluted tubules (DCT) and in the brain choroid plexus (Kuro-o *et al*, 1997). However, Klotho expression is also found at other locations, for example in renal proximal tubules (Hu *et al*, 2010; Andrukhova *et al*, 2012) or parathyroid glands (Shiraki-Iida *et al*, 1998; Urakawa *et al*, 2006; Imura *et al*, 2007). The molecular function of Klotho is still controversial. Klotho was initially thought to be an anti-aging factor (Kuro-o *et al*, 1997). Later studies suggested that soluble Klotho may have the ability to alter the function and abundance of membrane glycoproteins by removing sialic acid or other terminal sugars from sugar chains through a putative glycosidase activity (Chang *et al*, 2005; Kurosu *et al*, 2005; Hu *et al*, 2010).

Apart from its putative enzymatic function, membrane-bound Klotho also functions as the co-receptor for the bone-derived hormone fibroblast growth factor-23 (FGF23). Binding of FGF23 to ubiquitously expressed FGF receptors (FGFR) requires Klotho as an obligatory co-receptor (Kurosu *et al*, 2006; Urakawa *et al*, 2006), restricting the hormonal action of FGF23 to Klotho-expressing tissues. FGF23 down-regulates renal proximal tubular phosphate reuptake and 1α-hydroxylase expression. 1α-hydroxylase is the key enzyme for production of the biologically active vitamin D hormone, 1α,25-dihydroxyvitamin D_3_ (1,25(OH)_2_D). Because FGF23 is secreted by osteocytes and osteoblasts in bone in response to elevated phosphate and vitamin D, this hormone forms a negative feedback loop between bone and kidney (Juppner *et al*, 2010).

**Figure 1:**
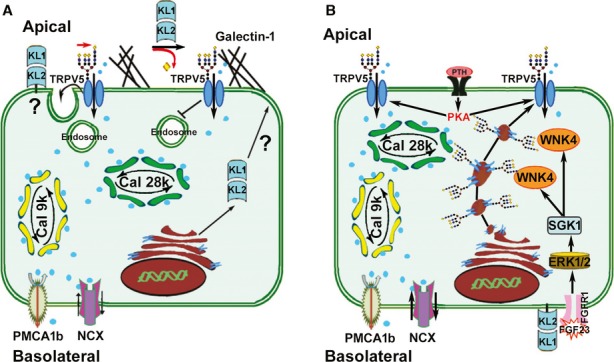
Models of the regulation of apical membrane TRPV5 in renal distal tubules by Klotho and FGF23.
Model based on previous studies for the regulation of apical membrane TRPV5 by secreted Klotho. TRPV5 is necessary for apical entry of calcium, which is then transported through the cell bound to calbindin D9k and D28k, and extruded at the basolateral side via PMCA1 and NCX. Secreted Klotho is thought to specifically hydrolyze sugar residues from the glycan chains on TRPV5 which in turn stabilizes TRPV5 in the membrane through interaction of the sugar residues with extracellular galectin (Chang *et al*, 2005; Cha *et al*, 2008). The cellular secretion process of Klotho in this model is unclear. Adapted from Cha *et al* (2008).Our proposed model of Fgf23-αKlotho signaling in renal distal tubular cells. Fgf23 binds to the basolateral FGFR1c-Klotho complex and activates ERK1/2 leading to SGK1 phosphorylation. SGK1 in turn activates WNK4, stimulating WNK4-TRPV5 complex formation, and increasing intracellular transport of fully glycosylated TRPV5 from the Golgi apparatus to the plasma membrane. PTH signaling activates membrane-anchored TRPV5 by protein kinase A (PKA)-mediated phosphorylation.

It was reported by Chang and colleagues (Chang *et al*, 2005) that soluble Klotho is a regulator of the epithelial calcium channel transient receptor potential vannilloid-5 (TRPV5), a glycoprotein essential for entry of calcium in calcium-transporting renal epithelial cells. Apical membrane expression of fully glycosylated TRPV5 is the rate-limiting step in distal renal tubular transcellular calcium transport which also involves the calcium-binding proteins calbindin D9k and D28k (CalD28k) for intracellular transport, and the sodium-calcium exchanger (NaCX) and the plasma membrane calcium ATPase 1b (PMCA1b) for basolateral extrusion of calcium (Lambers *et al*, 2006). The current model for TRPV5 regulation by Klotho is that soluble Klotho promotes insertion of TRPV5 in the apical membrane of distal tubular cells through its putative glycosidase activity, thereby stabilizing the interaction between glycosylated TRPV5 and membrane-bound galectin (Chang *et al*, 2005; Cha *et al*, 2008) (Fig 1A). A major explanatory gap in this model is how soluble Klotho crosses from the systemic circulation into the urinary space. It was reported that the 130 kDa isoform of Klotho protein can be detected in the urine of mice (Chang *et al*, 2005; Hu *et al*, 2010). The soluble forms of murine Klotho are large proteins with molecular weights of about 130 and 65 kDa for the shed and alternatively spliced isoforms, respectively (Shiraki-Iida *et al*, 1998; Imura *et al*, 2007). Because the glomerular filter is impermeable to proteins larger than approximately 60–65 kDa under physiological conditions, soluble Klotho in plasma cannot be filtered. Alternatively, Klotho could be secreted by distal tubular cells into the urinary space, or shed from the apical cell membrane. However, experimental evidence for tubular secretion or tubular apical membrane shedding of Klotho is lacking thus far.

In accordance with an important role of Klotho in the regulation of distal renal tubular TRPV5 activity, *Klotho* null *(Kl*^−/−^) mice on a vitamin D deficient diet lose urinary calcium despite unchanged renal expression of fully glycosylated TRPV5 (Alexander *et al*, 2009). However, this animal model is complicated by the fact that *Kl*^−/−^ mice have elevated 1,25(OH)_2_D blood levels. 1,25(OH)_2_D is thought to be involved in the regulation of distal tubular TRPV5 (Lambers *et al*, 2006). Due to the lacking suppressive effect of Fgf23 on renal 1α-hydroxylase activity, *Kl*^−/−^ and *Fgf23*^−/−^ mice produce excessive amounts of 1,25(OH)_2_D, and die early from the sequelae of hypervitaminosis D, hypercalcemia and hyperphosphatemia. The pivotal role of hypervitaminosis D for the development of the premature aging phenotype in *Kl*^−/−^ and *Fgf23*^−/−^ mice is underscored by the fact that ablation of vitamin D signaling completely rescues the premature aging phenotype in these mice (Hesse *et al*, 2007; Anour *et al*, 2012; Streicher *et al*, 2012).

In order to examine vitamin D independent effects of *Klotho* and *Fgf23* deficiency on renal calcium excretion in skeletally mature mice, we crossed mice with a non-functioning vitamin D receptor (VDR^Δ/Δ^) with *Kl*^−/−^ and *Fgf23*^−/−^ mice, and analyzed them at 9 months of age. All mice were kept life-long on a so-called rescue diet rich in calcium, phosphorus, and lactose. The rescue diet normalizes the calcium absorption defect of VDR mutant mice in the gut, so that VDR^Δ/Δ^ mice, but also *Kl*^−/−^/VDR^Δ/Δ^ and *Fgf23*^−/−^*/*VDR^*Δ/Δ*^ mice on this diet are normocalcemic (Erben *et al*, 2002; Hesse *et al*, 2007; Anour *et al*, 2012). In this study, we show that skeletally mature *Kl*^−/−^/VDR^Δ/Δ^ and *Fgf23*^−/−^*/*VDR^*Δ/Δ*^ mice are characterized by an almost identical renal calcium wasting phenotype, and that FGF23 is a regulator of distal tubular TRPV5 membrane abundance and renal calcium reabsorption through an intracellular signaling cascade involving ERK1/2, SGK1, and WNK4.

## Results

**Figure 2:**
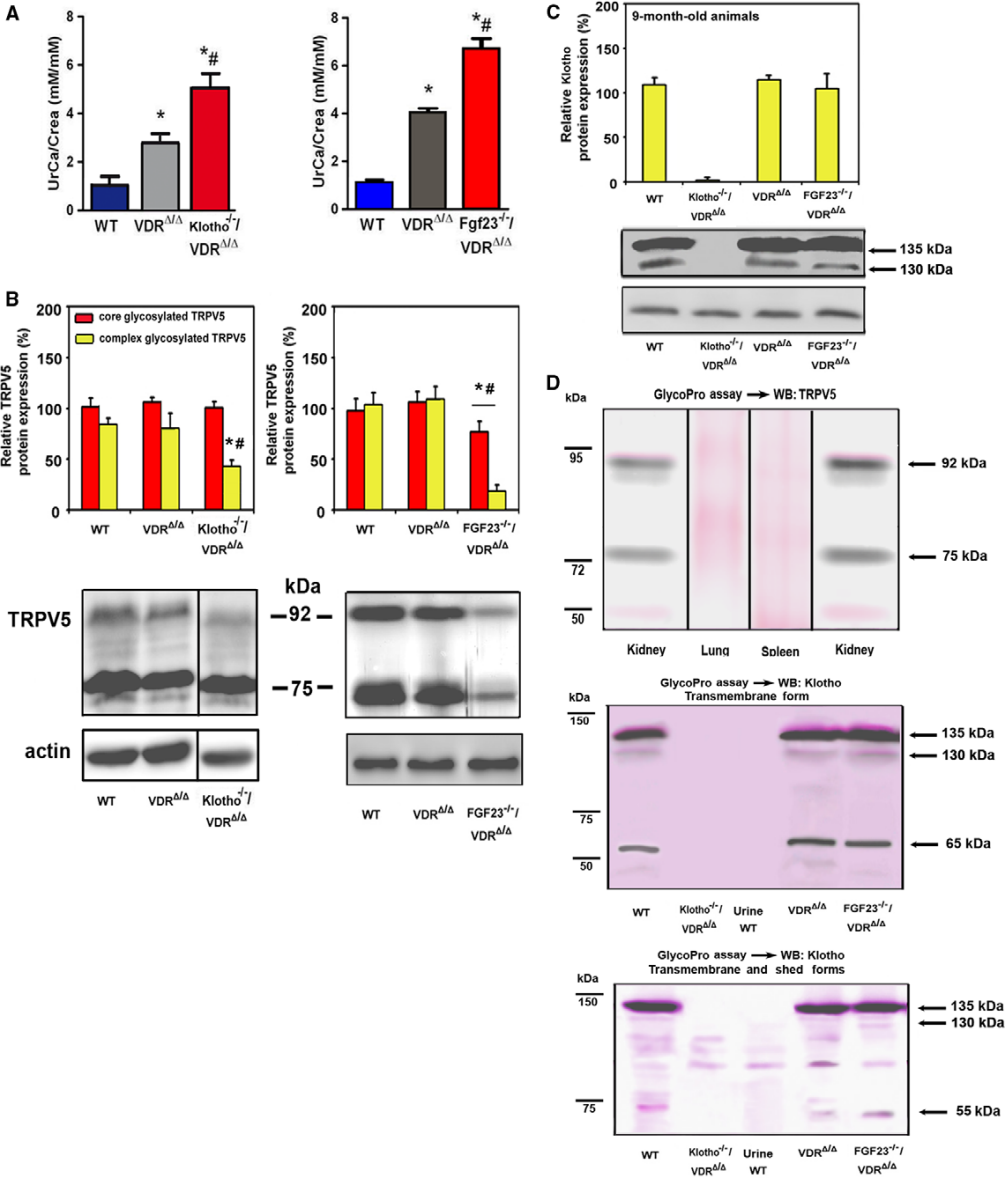
Renal calcium reabsorption and TRPV5 plasma membrane abundance in *Fgf23* and *Klotho* deficient mouse models. A–D Urinary excretion of calcium corrected for creatinine (UrCa/Crea) (A), Western blotting quantification of core (75 kDa) and complex (92 kDa) glycosylated TRPV5 protein expression in renal cortical total membrane fractions (B), and Western blot analysis of membrane-bound Klotho in renal total protein extracts (C) in 9-month-old male WT, VDR^Δ/Δ^, *Kl*^−/−^/VDR^Δ/Δ^, and *Fgf23*^−/−^/VDR^Δ/Δ^ on rescue diet. Antibody specificity and glycosylation of TRPV5 and Klotho was controlled by a glycosylation assay (pink staining) followed by Western blot analysis of TRPV5 and Klotho, respectively (D). Anti-Klotho antibodies detecting the membrane-bound (anti-cytoplasmic domain, upper panel) or the membrane-bound and ectodomain shed (anti KL2 domain, lower panel) forms of the protein were used. Protein extracts from lung and spleen, as well as kidney extracts from *Kl*^−/−^/VDR^Δ/Δ^ mice served as negative controls for TRPV5 and Klotho protein expression, respectively. Data information: *P < 0.05 vs. WT, ^#^*P* < 0.05 vs. VDR^Δ/Δ^ mice. Only the 135 kDa transmembrane isoform of Klotho was quantified in (C). Data in (A–C) represent mean ± s.e.m. of 4–9 animals each. Frames in Western blot images shown in (B) and (D) indicate splicing events. Source data are available online for this figure.

We first examined renal calcium excretion in skeletally mature, 9-month-old wild-type (WT), VDR^Δ/Δ^, and *Kl*^−/−^/VDR^Δ/Δ^ compound mutant mice on rescue diet. We found pronounced loss of urinary calcium in 9-month-old *Kl*^−/−^/VDR^Δ/Δ^ mice (Fig 2A), which was associated with a 50% downregulation of membrane expression of complex glycosylated TRPV5 relative to VDR^Δ/Δ^ mice (Fig 2B). It is known from previous studies that VDR mutant mice on rescue diet show a defect in renal tubular reabsorption of calcium due to lower expression of calbindin D9k (Erben *et al*, 2002). The core and complex glycosylated forms of TRPV5 protein were unchanged in VDR single mutant mice (Fig 2B), showing that vitamin D signaling is not essential for the regulation of TRPV5. However, urinary loss of calcium was almost twice as high in compound mutant mice compared with VDR single mutants, i.e., lack of *Klotho* aggravated the renal calcium wasting seen in VDR single mutants (Fig 2A). This finding corroborates earlier reports that Klotho has an essential role in the regulation of renal TRPV5 activity (Chang *et al*, 2005), and further shows that this mechanism is VDR independent. Notably, similar to *Kl*^−/−^/VDR^Δ/Δ^ mice, 9-month-old *Fgf23*^−/−^*/*VDR^*Δ/Δ*^ mice also showed renal calcium wasting and reduced membrane expression of TRPV5 (Fig 2A and B). Indeed, the absence of Fgf23 resulted in a stronger downregulation of core and complex glycosylated TRPV5 compared with the absence of Klotho (Fig 2B). Using anti-Klotho antibodies raised against the short intracellular region of the membrane-bound Klotho isoform or against the extracellular KL2 domain, we found renal Klotho protein expression unchanged in both VDR^*Δ/Δ*^ single and *Fgf23*^−/−^*/*VDR^*Δ/Δ*^ compound mutants (Fig 2C and Supplementary Fig S1A). Although the anti-TRPV5 and anti-Klotho antibodies we used for immunoblotting and immunohistochemistry have been successfully employed by other groups (Sandulache *et al*, 2006; Segawa *et al*, 2007), we confirmed the specificity of the antibodies, using tissue extracts from mouse lung and spleen where TRPV5 is not expressed (Fig 2D), extracts from kidneys of TRPV5^−/−^ mice (Supplementary Fig S1B), and extracts from kidneys of *Kl*^−/−^/VDR^Δ/Δ^ mice lacking all isoforms of Klotho (Fig 2C and D) as negative controls. In addition, we confirmed the glycosylation of the 92 kDa TRPV5 band and of the 135 kDa transmembrane form of Klotho (Fig 2D). Both anti-Klotho antibodies detected the 135 kDa glycosylated full-length transmembrane form as the predominant form of Klotho protein in renal extracts (Fig 2D). The 130 kDa band which might represent the ectodomain shed form of Klotho was also glycosylated (Fig 2D). The 65 kDa Klotho band detected by the anti-transmembrane antibody was not glycosylated, whereas the 55 kDa Klotho band detected by the anti-KL2 antibody was glycosylated (Fig 2D). Therefore, the nature of these two putative fragments of Klotho is not clear. Using the methodology described by Hu and coworkers (Hu *et al*, 2010), we were unable to detect any isoform of Klotho protein in native, salt-precipitated, or concentrated urine of WT mice by Western blotting (Fig 2D and Supplementary Fig S1C). The reason for this discrepancy is not clear, but may be due to differences in the anti-Klotho antibodies used.

**Figure 3:**
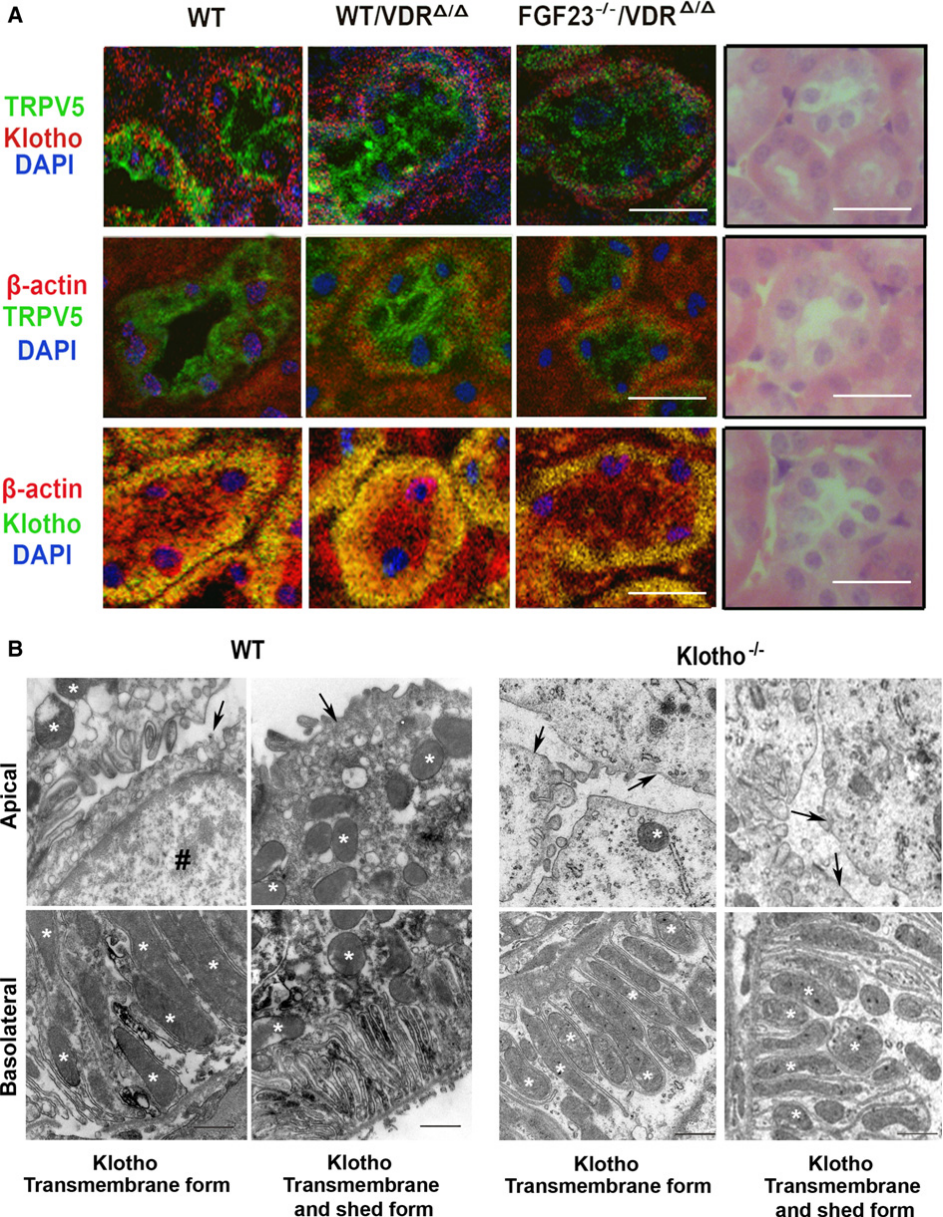
Membrane-bound αKlotho and TRPV5 do not co-localize in renal distal tubule cells.
Immunohistochemical co-staining with anti-transmembrane αKlotho (red or green) antibody, anti-TRPV5 (green), anti-β-actin (red), and DAPI (blue) of paraffin sections from kidneys of 9-month-old WT, VDR^Δ/Δ^, and *Fgf23*^−/−^/VDR^Δ/Δ^ mice on rescue diet. Right panels show H&E-stained paraffin sections for comparison of subcellular localization. Scale bar, 20 μm.Immuno-electron microscopic staining using anti-Klotho antibodies against transmembrane, and transmembrane and shed forms in kidneys of WT (left panels) and Klotho^−/−^ (right panels) mice. Upper panels show the apical cell area, lower panels show the basolateral area with the basal labyrinth. Arrows mark the apical cell membrane, asterisks mark mitochondria, and the symbol # marks the nucleus. Scale bar, 500 nm.

The fact that Klotho deficiency and Fgf23 deficiency have almost identical effects on renal TRPV5 is difficult to explain on the basis of the model shown in Fig 1A. Rather, this finding points to an essential role of Fgf23 in the regulation of TRPV5. We reported earlier that renal function and morphology of kidneys is normal in 9-month-old VDR^*Δ/Δ*^ and *Fgf23*^−/−^*/*VDR^*Δ/Δ*^ mice (Streicher *et al*, 2012), ruling out renal functional impairment as a confounding factor in these experiments. A central element of the model shown in Fig 1A is a direct, albeit transient, protein-protein interaction between TRPV5 and Klotho, because soluble Klotho is thought to enzymatically alter the glycosylation pattern of TRPV5 in the apical cell membrane (Chang *et al*, 2005). Co-localization of Klotho and TRPV5 in distal renal tubular cells has been demonstrated by Chang *et al* (Chang *et al*, 2005). In our immunohistochemical analysis, TRPV5 staining was exclusively seen in distal tubules also expressing calbindin D28k, further demonstrating the specificity of the antibody used (Supplementary Fig S2A). The membrane-bound form of Klotho was mainly found in the cytoplasm and the basolateral membrane, whereas TRPV5 was mainly localized in the apical membrane in WT, VDR^*Δ/Δ*^, and *Fgf23*^−/−^*/*VDR^*Δ/Δ*^ mice (Fig 3A). We observed an identical subcellular distribution of Klotho in distal tubular epithelium, employing an anti-Klotho antibody detecting both the membrane-bound and the ectodomain shed form of the protein (Supplementary Fig S2B). Some TRPV5 staining was also seen basolaterally in all genotypes (Fig 3A). Co-localization of Klotho and TRPV5, however, was almost absent, and only seen in some cytoplasmic or basolateral areas of the distal tubular cells (Fig 3A and Supplementary Fig S2). In analogy to the immunoblotting data (Fig 2B), membrane expression of TRPV5 was clearly reduced in distal tubules of *Fgf23*^−/−^*/*VDR^*Δ/Δ*^ mice (Fig 3A). To assess the subcellular localization of Klotho in more detail, we performed immuno-electron microscopic analyses in renal tissue from WT mice, using anti-Klotho antibodies detecting either the transmembrane or both the transmembrane and the ectodomain shed forms of the protein. Both antibodies showed the presence of Klotho protein in the membrane of the basal labyrinth, but staining was absent in the apical membrane of distal tubular cells (Fig 3B). Kidneys from *Kl*^−/−^ mice were used as a negative control (Fig 3B).

**Figure 4:**
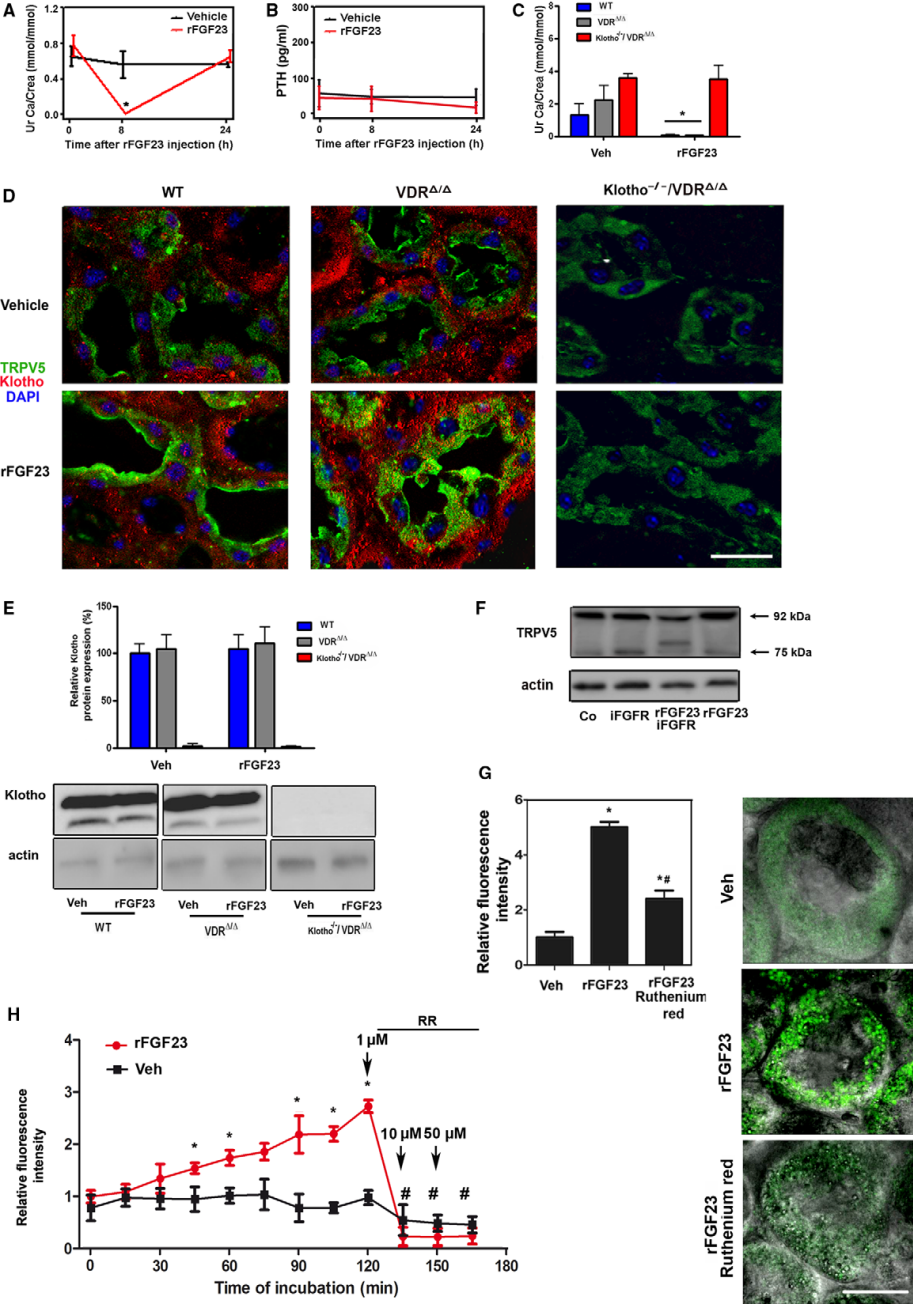
FGF23 increases urinary calcium reabsorption, TRPV5 plasma membrane abundance and activity in the kidney in gain-of-function mouse models. A, B Urinary calcium excretion (A) and serum PTH (B) in 4-month-old WT mice injected i.p. with vehicle or a single dose of 10 μg rFGF23 per mouse at time 0. C Urinary calcium excretion in 4-month-old WT, VDR^Δ/Δ^, and *Kl*^−/−^/VDR^Δ/Δ^ mice on rescue diet injected i.p. with vehicle or a single dose of 10 μg rFGF23 per mouse, 8 h post-injection. D Immunohistochemical co-staining of kidney paraffin sections with anti-transmembrane αKlotho (red) antibody, anti-TRPV5 (green) and DAPI (blue). Original magnification ×630. E Western blot analysis of membrane-bound Klotho in renal total protein extracts from 4-month-old WT, VDR^Δ/Δ^, and *Kl*^−/−^/VDR^Δ/Δ^ mice treated with vehicle or rFGF23 (10 μg/mouse) 8 h before necropsy. F Complex glycosylated TRPV5 protein expression in isolated distal tubular segments treated for 2 h *in vitro* with rFGF23 alone or in combination with a specific FGFR inhibitor (iFGFR). G Quantification and original images of intracellular Ca^2+^ levels in renal distal tubular cells in 300-μm-thick kidney slices of 3-month-old WT mice treated with vehicle or rFGF23 (10 μg/mouse) 8 h before necropsy. Images are overlays of fluorescent with phase contrast images. Kidney slices were stained with the calcium-sensitive dye Fluo-4. Ruthenium red (10 μM) was used as TRPV inhibitor. H Time-dependent changes in distal tubular fluorescence in Fluo-4-loaded, 300-μm-thick kidney slices of 3-month-old WT mice treated at time 0 with rFGF23 (100 ng/ml) or vehicle. After 120, 135, and 150 min, 1, 10, and 50 μM of the TRPV inhibitor ruthenium red (RR) was added, respectively. Data information: Data in (A–C) represent mean ± s.e.m. of 3–5 animals each. *P < 0.05 vs. vehicle-treated control in (A–C). Data in (E) represent mean ± s.e.m. of 3–5 animals each. In (G), *P < 0.05 vs. vehicle-treated, ^#^*P* < 0.05 vs. rFGF23-treated WT mice. In (H), *P < 0.05 vs. vehicle, ^#^*P* < 0.05 vs. fluorescence at 120 min in rFGF23-treated slices. Fluorescence intensity in (G) and (H) was quantified in 7–9 regions of interest per experimental group and time point from three independent experiments. Scale bar, 20 μm in (D) and (G). Source data are available online for this figure.

To examine whether FGF23 was able to regulate TRPV5 in gain-of-function models, we treated WT, VDR^Δ/Δ^, and *Kl*^−/−^/VDR^Δ/Δ^ mice with recombinant FGF23 (rFGF23). Within 8 h post-injection, rFGF23 reduced urinary calcium excretion to very low levels in WT mice (Fig 4A). Serum calcium and serum parathyroid hormone (PTH) remained unchanged (Supplementary Fig S3A and Fig 4B). The rFGF23-induced reduction in urinary calcium excretion occurred in WT and VDR^Δ/Δ^, but not in *Kl*^−/−^/VDR^Δ/Δ^ mice (Fig 4C), and was associated with distinctly upregulated TRPV5 expression in the distal tubular apical membrane (Fig 4D) of WT and VDR^Δ/Δ^, but not of *Kl*^−/−^/VDR^Δ/Δ^ mice. These data demonstrate that the FGF23-induced upregulation of TRPV5 and the concomitant increase in renal tubular calcium reabsorption are Klotho dependent. Despite the profound changes in TRPV5 expression and renal calcium reabsorption in WT and VDR^Δ/Δ^ mice, expression of membrane-bound and shed Klotho protein remained unchanged (Fig 4E and Supplementary Fig S3B), and Klotho did not show co-localization with TRPV5 in the distal tubular apical membrane (Fig 4D and Supplementary Fig S3C). Immunohistochemical Klotho staining was absent in *Kl*^−/−^/VDR^Δ/Δ^ mice (Fig 4D and Supplementary Fig S3C), confirming the specificity of both anti-Klotho antibodies used. Moreover, soluble Klotho in serum remained below the detection limit of an immunoassay specific for murine Klotho (data not shown) and anti-Klotho immunoblotting of urine was negative in rFGF23-treated WT mice (Supplementary Fig S3D). Finally, we treated isolated distal tubular segments *in vitro* with rFGF23 in the presence and absence of a FGFR inhibitor. The FGF23-induced upregulation of complex glycosylated TRPV5 expression was completely blunted in the presence of the FGFR inhibitor, showing that FGF23 signals through the FGFR to increase distal tubular TRPV5 membrane expression (Fig 4F).

To confirm the functional role of the FGF23-induced upregulation of TRPV5 in the apical membrane of distal tubular epithelium, we performed intracellular calcium imaging, employing 2-photon microscopy of Fluo-4-loaded, 300-μm-thick renal slices prepared from vehicle-and rFGF23-treated WT mice, 8 h post-injection. Distal tubules of FGF23-treated mice showed a 5-fold increase in fluorescence intensity, relative to those of vehicle-treated mice (Fig 4G). The FGF23-induced increase in fluorescence intensity was largely abrogated by *ex vivo* addition of 10 μM of the TRPV inhibitor ruthenium red (Fig 4G). Supplementary videos 1 and 2 show the changes in fluorescence over time (30 min) after addition of ruthenium red in kidney slices from rFGF23-and vehicle-treated mice, respectively. For distal tubular calcium reabsorption, only TRPV5 and 6 are thought to be relevant (Woudenberg-Vrenken *et al*, 2009). TRPV5 is about 100-fold more sensitive to ruthenium red than TRPV6 (Hoenderop *et al*, 2001). Therefore, ruthenium red can be used to discriminate the channel function of TRPV5 from that of TRPV6. To further demonstrate the regulation of TRPV5 activity by FGF23, we treated Fluo-4-loaded kidney slices from WT mice with rFGF23 or vehicle *in vitro*. rFGF23 gradually increased intracellular fluorescence over 2 h in distal tubules (Fig 4H and Supplementary video 3). The latter effect was reversed by ruthenium red at concentrations as low as 1 μM (Fig 4H and Supplementary video 4). At 1 μM, ruthenium red does not antagonize TRPV6 function (Hoenderop *et al*, 2001). 10 and 50 μM of ruthenium red did not further decrease the fluorescence signal, showing that TRPV6 does not play a major role in the FGF23-induced increase in calcium uptake in distal tubular cells in this experimental setting (Fig 4H and Supplementary video 4). The discrepancy between the complete reversal of the rFGF23-induced increase in intracellular Fluo-4 fluorescence by ruthenium red in Fig 4H and the partial reversal observed in Fig 4G can probably be explained by the fact that in the *ex vivo* experiment shown in Fig 4G distal tubular cells were pre-exposed to rFGF23 for 8 h, whereas rFGF23 was added at time 0 in the *in vitro* experiment shown in Fig 4H. The latter experimental setting therefore looks at very early aspects of the rFGF23-induced increase in distal tubular calcium uptake. This is also reflected in the almost 2-fold higher rFGF23-induced increase in relative fluorescence in the *ex vivo* (Fig 4G) compared with the *in vitro* experiment (Fig 4H). Collectively, these data show that the FGF23-induced upregulation of TRPV5 results in increased calcium uptake in distal tubular epithelial cells, and that the FGF23-induced stimulation of apical calcium entry is mainly mediated by TRPV5. However, because ruthenium red at 10 μM did not completely reverse the rFGF23-induced increase in intracellular Fluo-4 fluorescence in distal tubules (Fig 4G) and TRPV5 and TRPV6 can form heteromultimers (Hellwig *et al*, 2005) with intermediate ruthenium red sensitivity, we cannot exclude a potential contribution of TRPV6 to the effects of FGF23 on renal tubular calcium reabsorption *in vivo*.

The intracellular calcium-binding protein calbindin D28k is thought to be involved in intracellular calcium transport in renal distal epithelium (Lambers *et al*, 2006). Therefore, we examined the expression and intracellular distribution of this cytoplasmic protein in kidneys of vehicle-or rFGF23-treated WT mice. We found upregulated mRNA abundance, a trend towards increased protein expression, but no change in the intracellular distribution pattern of calbindin D28k, 8 h after rFGF23 administration (Supplementary Fig S3E and F).

In summary, these data demonstrate that FGF23 regulates renal TRPV5 in loss-and gain-of-function models independent of alterations in Klotho expression; Klotho is mainly expressed basolaterally in distal tubule epithelium and does not co-localize with TRPV5 in the apical cell membrane; soluble Klotho is undetectable in urine, and FGF23 signals through the FGFR-Klotho complex to regulate TRPV5 in distal tubules. These findings do not support the signaling model shown in Fig 1A. Rather, the data suggest that FGF23 regulates membrane abundance of TRPV5 by a signaling mechanism in which Klotho functions as the co-receptor required for FGF23 signaling. Hence, we next asked how FGF23 signaling influenced distal tubular membrane abundance of TRPV5.

FGF23 signaling leads to activation of extracellular signal-regulated kinase 1 and 2 (ERK1/2) (Urakawa *et al*, 2006) which itself activates serum and glucocorticoid-induced kinase 1 (SGK1) in renal mouse cortical collecting duct cells (Michlig *et al*, 2004), and in renal proximal tubules (Andrukhova *et al*, 2012). Renal DCT SGK1 phosphorylates and thereby activates with-no-lysine kinase 4 (WNK4) (Ring *et al*, 2007), which is critically involved in membrane transport of other ion channels in the DCT such as Na^+^-Cl^−^ co-transporter (NCC) and renal outer medullar K^+^channel (ROMK1) (Ring *et al*, 2007). WNK4-mediated regulation of these channels involves physical association between the channel protein and WNK4 (Cai *et al*, 2006; He *et al*, 2007). Interestingly, mutations in WNK4 can lead to urinary calcium loss in humans (Mayan *et al*, 2002). In addition, studies in transfected *Xenopus laevis* oocytes have suggested that WNK4 influences the membrane abundance of TRPV5 (Jiang *et al*, 2007, 2008).

**Figure 5:**
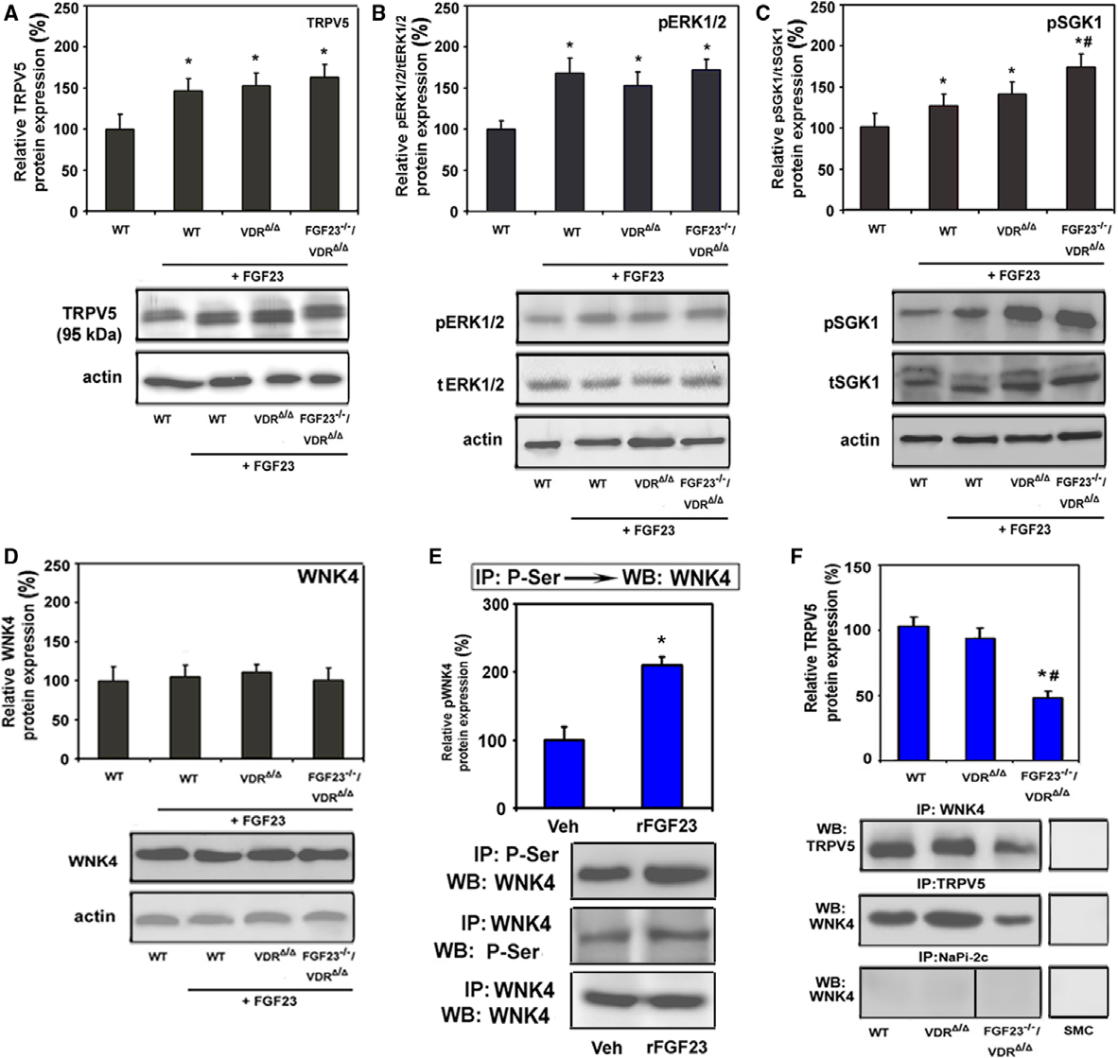
FGF23 increases TRPV5 protein abundance in the plasma membrane through a signaling pathway involving ERK1/2, SGK1, and WNK4. A–D Western blot analysis of complex glycosylated TRPV5 (A) protein (92 kDa) expression in renal cortical total membrane fractions, as well as phosphorylated ERK1/2 (B), phosphorylated SGK1 (C), and total WNK4 (D) in homogenized renal cortex samples of 9-month-old WT, VDR^Δ/Δ^ and *Fgf23*^−/−^/VDR^Δ/Δ^ mice on rescue diet that had been injected with rFGF23 (5 μg/mouse). Tissue samples were taken from mice 8 h post-injection. E Reciprocal immunoprecipitation (IP) of serine-phosphorylated (P-Ser) proteins, followed by Western blot (WB) analysis of WNK4 or vice versa from homogenized renal cortex protein samples of 4-month-old WT mice treated with vehicle (Veh) or rFGF23 (10 μg/mouse) 8 h before necropsy. Western blot analysis of WNK4 in renal cortex protein samples immunoprecipitated with WNK4 antibody was used as a loading control. F Co-immunoprecipitation of TRPV5/WNK4 complexes. WNK4 or TRPV5 were immunoprecipitated with specific antibodies (anti-TRPV5, anti-WNK4, anti-NaPi-2c) from homogenized renal cortex protein samples of 9-month-old WT, VDR^Δ/Δ^ and *Fgf23*^−/−^/VDR^Δ/Δ^ mice on rescue diet. Subsequently, Western blot analysis was performed with corresponding anti-TRPV5 or anti-WNK4 antibodies to identify co-precipitated TRPV5 and WNK4 protein, respectively. Primary cultured murine smooth muscle cells (SMC) were used as negative control. Data information: Data in (A–D) represent mean ± s.e.m. of 5–8 animals each, *P < 0.05 vs. vehicle-treated WT, ^#^*P* < 0.05 vs. rFGF23-treated WT mice. Data in (E) represent mean ± s.e.m. of five animals each. Data in (F) represent mean ± s.e.m. of 4–5 animals each. Frame in Western blot image indicates splicing event. In (F) *P < 0.05 vs. WT, ^#^*P* < 0.05 vs. VDR^Δ/Δ^ mice. Source data are available online for this figure.

Based on this knowledge, we hypothesized that FGF23 may regulate TRPV5 through a signaling cascade involving ERK1/2, SGK1, and WNK4. To test this, we first examined whether activation of ERK1/2 and SGK1 was regulated by FGF23 in loss-and gain-of-function models. Phospho-ERK1/2 and phospho-SGK1 were decreased in the kidney of *Fgf23*^−/−^*/*VDR^*Δ/Δ*^ compound mutant mice (Supplementary Fig S4A and B). rFGF23 administration to WT, VDR^*Δ/Δ*^, and *Fgf23*^−/−^*/*VDR^*Δ/Δ*^ compound mutant mice led to an increase in renal complex glycosylated TRPV5 (Fig 5A), phospho-ERK1/2 (Fig 5B), and phospho-SGK1 (Fig 5C). These findings suggest that FGF23 signaling leads to activation of renal SGK1 and increased membrane abundance of complex glycosylated TRPV5 in a VDR independent fashion.

Next, we examined whether WNK4 is a downstream mediator of FGF23 signaling. Reciprocal immunoprecipitation experiments on renal protein extracts, using anti-phosphoserine and anti-WNK4 antibodies showed that serine phosphorylation of WNK4 was increased 8 h after treatment of WT mice with rFGF23 (Fig 5E). Further, reciprocal immunoprecipitation revealed a physical association between fully glycosylated TRPV5 and WNK4 in the kidney of WT, VDR^*Δ/Δ*^ and *Fgf23*^−/−^*/*VDR^*Δ/Δ*^ mice (Fig 5F). Whereas total WNK4 protein abundance was not different between the genotypes (Fig 5D), the amount of TRPV5-WNK4 complexes in kidneys of *Fgf23*^−/−^*/*VDR^*Δ/Δ*^ mutant mice was decreased by about 50% (Fig 5F). As a control for the specificity of the co-immunoprecipitation, we used an antibody against the sodium phosphate transporter-2c (NaPi-2c), a protein exclusively expressed in renal proximal tubules. As an additional specificity control, the co-immunoprecipitation experiments were also done using protein extracts from cultured murine smooth muscle cells, which neither express TRPV5 nor WNK4 (Fig 5F).

**Figure 6:**
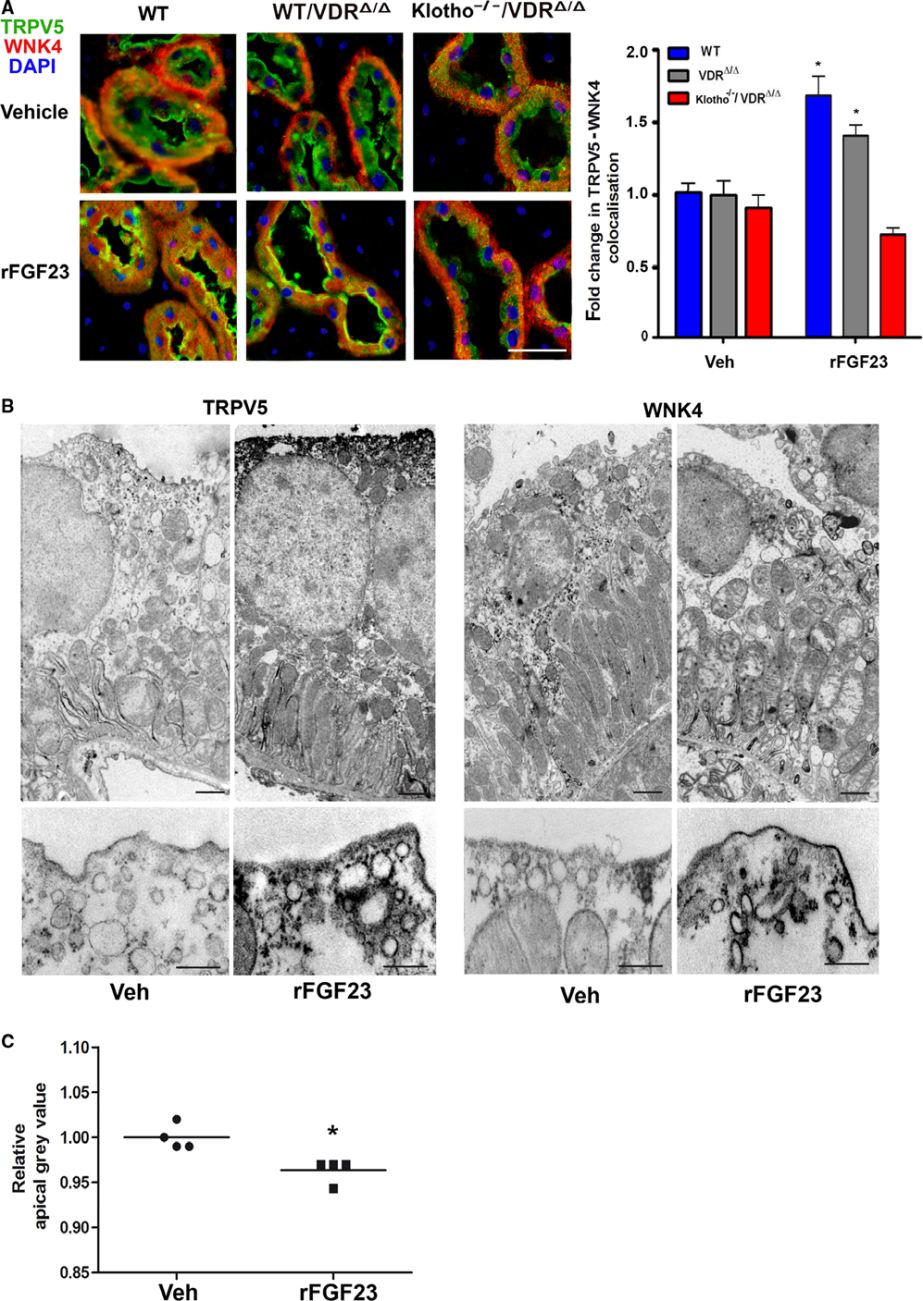
FGF23 increases apical plasma membrane abundance of TRPV5 and causes re-distribution of WNK4.
Immunohistochemical co-staining with anti-WNK4 (red) antibody, anti-TRPV5 (green) and DAPI (blue) of kidney paraffin sections from 4-month-old WT, VDR^Δ/Δ^ and Klotho^−/−^/VDR^Δ/Δ^ mice treated with vehicle or rFGF23 (10 μg/mouse) 8 h before necropsy. Scale bar, 20 μm. Right panel shows quantification of distal tubular TRPV5 and WNK4 co-localization performed in four animals per group with 3–6 images per animal and 4–6 different regions of interest per image.Immuno-electron microscopic staining using anti-TRPV5 (left panels) and anti-WNK4 (right panels) antibodies in kidneys from 3 to 4-month-old WT mice treated with vehicle (Veh) or rFGF23 (10 μg/mouse) 8 h before necropsy. Lower panels show higher magnification of the apical cell area from representative sections. Scale bar, 500 nm.Quantification of the relative grey levels in the apical area of the distal tubular cells between nucleus and apical cell membrane in the anti-TRPV5-stained immuno-electron microscopic pictures performed in four animals per group with 4–5 images per animal and 4–6 different regions of interest per image. Data information: *P < 0.05 vs. vehicle-treated controls in (A) and (C).

As far as it is known, WNK4 mainly regulates the trafficking of proteins from the Golgi apparatus to the plasma membrane and their glycosylation (Jiang *et al*, 2007, 2008). However, WNK4 may also be involved in regulating TRPV5 endocytosis (Cha & Huang, 2010). Immunohistochemical analysis suggested a subcellular redistribution of WNK4 from the basolateral side of the distal tubular cells to the area beneath the apical cell membrane in rFGF23-treated WT and VDR^Δ/Δ^, but not *Kl*^−/−^/VDR^Δ/Δ^ mice (Fig 6A). In addition, WNK4 and TRPV5 appeared to co-localize after rFGF23 treatment beneath, but not directly within, the apical cell membrane in WT and VDR^Δ/Δ^ mice (Fig 6A). Quantitative analysis of the immunohistochemical images confirmed the increase in TRPV5-WNK4 co-localization after rFGF23 treatment in WT and VDR^Δ/Δ^, but not *Kl*^−/−^/VDR^Δ/Δ^ mice (Fig 6A).

To assess the FGF23-induced changes in subcellular distribution of TRPV5 and WNK4 in more detail, we performed immuno-electron microscopic analyses in renal tissue taken from WT mice 8 h after a single injection of rFGF23. TRPV5 immunostaining was found exclusively in distal tubules and not in proximal tubules (Supplementary Fig S5). In vehicle controls, TRPV5 was mainly found in the apical cell membrane and the basal labyrinth in distal tubular cells, with some TRPV5 present in membrane vesicles (Fig 6B). FGF23 treatment induced a striking increase in TRPV5 present in the apical cell membrane as well as in membrane vesicles trafficking from the Golgi apparatus to the apical plasma membrane (Fig 6B). Quantification of the relative grey levels in the apical area of the distal tubular cells between nucleus and apical cell membrane in the immuno-electron microscopic pictures showed reduced grey levels (i.e., more black) in rFGF23-treated mice (Fig 6C), in accordance with increased abundance of TRPV5-containing membrane vesicles in the sub-apical cellular area.

In vehicle controls, WNK4 was mainly associated with localized areas in the basal labyrinth as well as with membrane vesicles trafficking to the apical plasma membrane, with most of the immunostaining observed in the peri-nuclear area (Fig 6B). Eight hours after rFGF23 treatment, WNK4 was still found at the basal labyrinth, but the main localization was around membrane vesicles in close proximity to and along the apical plasma membrane (Fig 6B). These findings are consistent with the notion that activated WNK4 is involved in the FGF23-induced regulation of the cellular trafficking of TRPV5 from the Golgi apparatus to the apical plasma membrane in distal tubular epithelium, especially in fusion of membrane vesicles with the apical cell membrane. We found no evidence of WNK4 associated with endocytotic pits.

**Figure 7:**
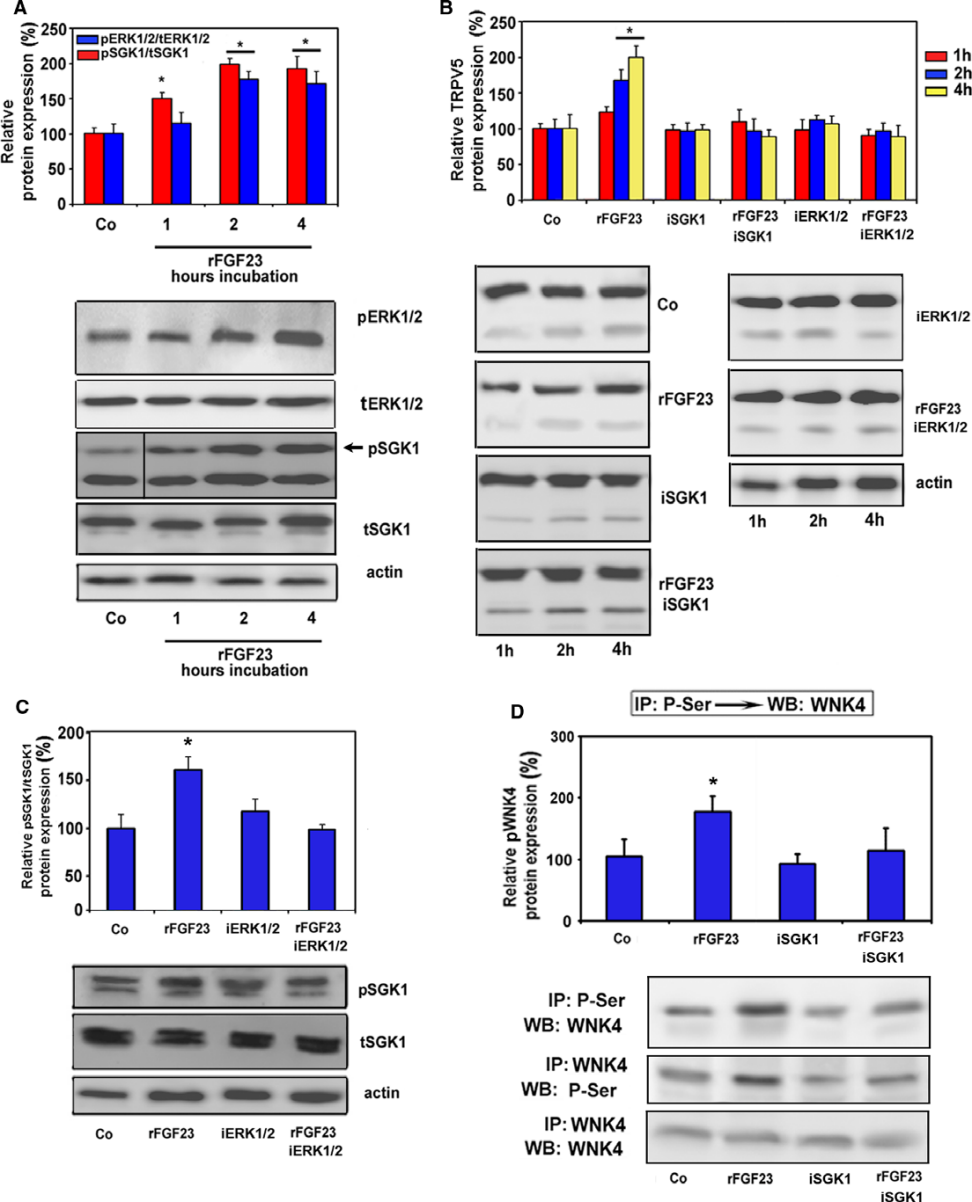
FGF23-induced increase in TRPV5 protein abundance in the plasma membrane is mediated by a signaling cascade involving ERK1/2 and SGK1. A, B Phosphorylated ERK1/2 and SGK1 (A) and complex glycosylated TRPV5 (B). Protein expression in isolated distal tubular segments from WT mice treated *in vitro* with rFGF23 alone or in combination with specific SGK1 (iSGK1) and ERK1/2 (iERK1/2) inhibitors. Frame in Western blot image in (A) indicates splicing event. C Phosphorylated SGK1 in isolated distal tubular segments treated *in vitro* with rFGF23 alone or in combination with a specific ERK1/2 inhibitor (iERK1/2). D Reciprocal immunoprecipitation (IP) of serine-phosphorylated (P-Ser) proteins, followed by Western blot (WB) analysis of WNK4 or vice versa from isolated distal tubular segments treated *in vitro* with rFGF23 alone or in combination with a specific SGK1 inhibitor (iSGK1). Western blot analysis of WNK4 in renal cortex protein samples immunoprecipitated with WNK4 antibody was used as a loading control. Data information: Actin loading controls are shown for tSGK1 in (A), rFGF23 + iERK1/2 in (B), and tSGK1 in (C). All presented protein expression values were normalized to individual actin levels. Data in (A–C) represent mean ± s.e.m. of 4–5 samples each. Data in (D) represent mean ± s.e.m. of 3–4 samples each. *P < 0.05 vs. vehicle control (Co). Source data are available online for this figure.

To verify that the regulation of TRPV5 by FGF23 is a direct effect on the distal tubule and to test the essential roles of ERK1/2 and SGK1 in this process, we isolated distal tubular segments from WT mice, and treated these segments with rFGF23 alone or rFGF23 together with specific SGK1 or ERK1/2 inhibitors for 1 to 4 h *in vitro*. rFGF23 time-dependently upregulated phosphorylation of ERK1/2 and SGK1 (Fig 7A), as well as protein expression of TRPV5 in distal tubular segments (Fig 7B). The latter effect was completely blocked in the presence of SGK1 or ERK1/2 inhibitors (Fig 7B), showing that ERK1/2 and SGK1 activation are essential for the FGF23-induced regulation of TRPV5 expression in the distal tubule. In the presence of an ERK1/2 inhibitor, rFGF23 did not increase phosphorylation of SGK1 in isolated proximal tubular segments from WT mice, indicating that SGK1 is a mediator downstream of ERK1/2 activation (Fig 7C). Furthermore, immunoprecipitation experiments in isolated distal tubular segments from WT mice revealed that the rFGF23-induced increase in serine phosphorylation of WNK4 is blocked in the presence of an SGK1 inhibitor (Fig 7D). Thus, activation of ERK1/2 and SGK1 by FGF23 leads to downstream activation of WNK4.

In order to explore the contribution of other pathways initiated by FGF23 signaling through the FGFR-Klotho complex, we treated isolated distal tubular segments with rFGF23 or with specific Src kinase (SRC), phospholipase C (PLC), and phosphatidylinositol 3-kinase (PI3K) inhibitors, alone or in combination, for 2 h *in vitro*. SRC and PLC inhibition did not influence the rFGF23-induced upregulation of TRPV5 expression (Supplementary Fig S6). However, PI3K inhibition partially blocked the rFGF23-induced increase in TRPV5 expression (Supplementary Fig S6). Taken together, these data suggest that ERK1/2 activation is the major pathway, but that the PI3K pathway may play an additional role in the regulation of TRPV5 by FGF23 in renal distal tubules.

**Figure 8:**
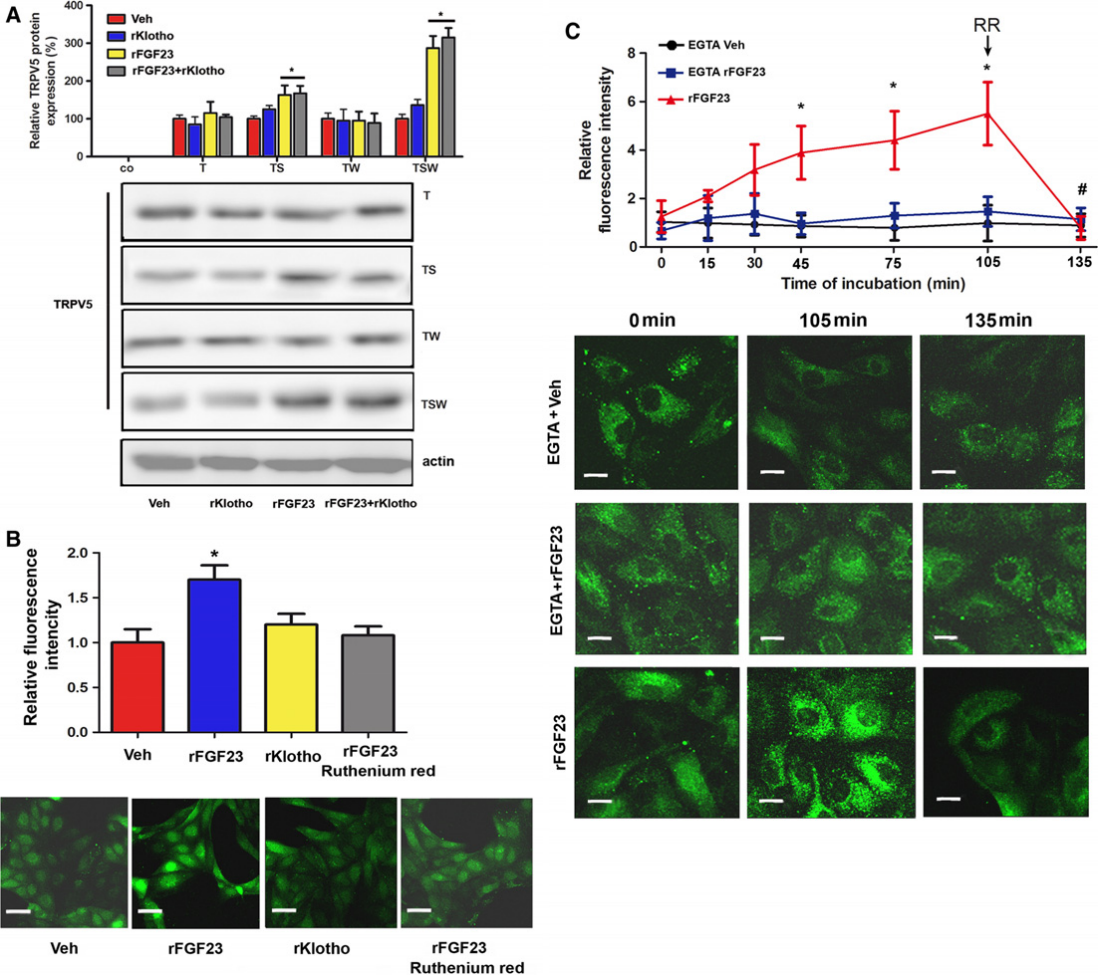
FGF23 increases TRPV5 protein abundance and channel activity in MDCK cells transfected with murine TRPV5, SGK1 and WNK4.
Complex glycosylated TRPV5 protein expression in total protein homogenates of MDCK cells transiently transfected with murine TRPV5 (T), SGK1 (S) and WNK4 (W) constructs, alone or in combination, and treated for 12 h with vehicle, rFGF23 or recombinant Klotho (rKlotho) alone or in combination. Mock-transfected cells were used as a negative control (Co).Quantification and original images of intracellular Ca^2+^ levels in MDCK cells transiently transfected with murine TRPV5, SGK1 and WNK4 constructs after treatment with vehicle, rFGF23 or recombinant Klotho (rKlotho) for 12 h. Ruthenium red (10 μM) was used as TRPV inhibitor.Time-dependent changes and original images of intracellular Ca^2+^ levels in MDCK cells transiently transfected with murine TRPV5, SGK1 and WNK4 constructs, and treated at time 0 with rFGF23 (100 ng/ml) or vehicle in the presence or absence of 1.5 mM EGTA added to the culture medium. After 105 min, 1 μM of ruthenium red (RR) was added. MDCK cells were stained with the calcium-sensitive dye Fluo-4 in (B) and (C). Data information: Data in (A) represent mean ± s.e.m. of 6–9 samples each from three independent experiments. Data in (B) and (C) represent mean ± s.e.m. of 3–4 samples each from three independent experiments. Scale bar, 20 μm in (B), 7 μm in (C). *P < 0.05 vs. vehicle in (A) and (B), and vs. EGTA + rFGF23 in (C), ^#^*P* < 0.05 vs. fluorescence at 105 min in (C). Source data are available online for this figure.

In order to more rigorously establish the putative FGFR-ERK1/2-SGK1-WNK4 signaling pathway of the TRPV5 regulation by FGF23, we reconstituted this pathway in MDCK cells. To this end, we transfected MDCK cells with murine TRPV5, SGK1, and WNK4 alone or in combination, treated the transfected cells with vehicle or rFGF23 alone or in combination with soluble Klotho for 12 h, and subsequently examined complex glycosylated TRPV5 protein expression by immunoblotting. It is known that MDCK cells express ERK1/2 and FGFR1 (Kessler *et al*, 2008), and as shown in Fig 8A, these cells do not endogenously express TRPV5. rFGF23 profoundly upregulated TRPV5 expression in MDCK cells co-transfected with TRPV5, SGK1, and WNK4 (Fig 8A). Recombinant soluble Klotho protein, alone or in combination with rFGF23, did not influence TRPV5 expression (Fig 8A). In the absence of SGK1, rFGF23 failed to upregulate TRPV5 expression (Fig 8A). However, small increases in TRPV5 expression in response to rFGF23 treatment were seen in cells transfected with TRPV5 and SGK1 only (Fig 8A). A possible explanation for this finding may be that MDCK cells, although not reported in the literature, might express low amounts of WNK4 or other WNK kinases. To examine the biological response to the rFGF23-induced increase in TRPV5 expression, we transfected MDCK cells with TRPV5, SGK1, and WNK4, treated the transfected cells with vehicle, rFGF23 or Klotho for 12 h, and then monitored intracellular calcium concentrations by 2-photon microscopy after loading the cells with Fluo-4. rFGF23 induced a striking increase in intracellular calcium in MDCK cells transfected with TRPV5, SGK1 and WNK4, reflecting increased TRPV5 channel activity (Fig 8B). In contrast, recombinant soluble Klotho protein did not influence intracellular calcium in TRPV5-expressing cells (Fig 8B). As expected, the rFGF23-induced increase in intracellular calcium could be blocked by the TRPV inhibitor ruthenium red (Fig 8B). To unequivocally show that FGF23 stimulates a TRPV5-mediated calcium influx pathway, we treated MDCK cells transfected with TRPV5, SGK1 and WNK4 with rFGF23 in the presence or absence of the calcium chelator EGTA (Fig 8C). In the presence of EGTA, rFGF23 did not increase intracellular Fluo-4 fluorescence (Fig 8C). These data firmly establish that the SGK1-WNK4 pathway mediates the regulation of TRPV5 expression and activity by FGF23 in renal epithelial cells, and further suggest that soluble Klotho does not influence TRPV5 channel activity in these cells.

## Discussion

Our data have uncovered a previously unknown VDR independent function of FGF23 signaling in renal calcium transport. We propose the model shown in Fig 1B which is able to explain the striking similarities between *Kl*^−/−^/VDR^Δ/Δ^ and *Fgf23*^−/−^*/*VDR^*Δ/Δ*^ mice in the regulation of TRPV5. Klotho functions as the co-receptor for blood-borne FGF23 in this model, not as a calcium-regulating hormone *per se*. In analogy to the other major phosphaturic hormone PTH, FGF23 is not only a phosphaturic, but also a calcium-conserving hormone in the kidney, activating, through the FGFR-Klotho complex, a signaling cascade involving ERK1/2, SGK1, and WNK4. Thus, our data may explain the hitherto unresolved question why injection of FGF23 does not lower serum calcium despite its suppressive action on renal synthesis of bioactive vitamin D (Shimada *et al*, 2001, 2004).

Although several reports have suggested that soluble Klotho may regulate the membrane abundance and function of ion channels such as TRPV5, ROMK and NaPi-2a in an FGF23-independent manner, and likewise the function of receptors such as the IGF-1 receptor (Chang *et al*, 2005; Kurosu *et al*, 2005; Hu *et al*, 2010), there is accumulating evidence that Klotho does not have FGF23-independent effects on mineral or glucose homeostasis *in vivo*. For example, the phenotype of *Fgf23* and *Klotho* double knockout mice is indistinguishable from that of *Fgf23*^−/−^ and *Kl*^−/−^ single knockout mice (Nakatani *et al*, 2009), strongly arguing against FGF23-independent functions of Klotho. In addition, we previously showed that glucose metabolism, bone turnover, and steady-state PTH secretion is unchanged in *Kl*^−/−^/VDR^Δ/Δ^ compared with VDR^Δ/Δ^ mice (Anour *et al*, 2012), suggesting that Klotho does not have physiologically relevant VDR-and Fgf23-independent functions in the regulation of mineral and glucose homeostasis *in vivo*. Thus, the major physiological role of Klotho appears to be its function as an obligatory co-receptor for FGF23. However, there is no conclusive evidence at present to rule out an additional enzymatic function of Klotho.

To address the question of whether or not Klotho has FGF23-independent functions on calcium metabolism, we attempted breeding 4-week-old *Fgf23*^−/−^*/Kl*^−/−^/VDR^Δ/Δ^ triple mutant mice on rescue diet in order to compare the phenotype of these mice to that of *Fgf23*^−/−^/VDR^Δ/Δ^ and *Kl*^−/−^/VDR^Δ/Δ^ double mutant mice. The breeding yielded only one *Fgf23*^−/−^*/Kl*^−/−^*/*VDR^Δ/Δ^ triple mutant animal so far. This animal displayed no difference in gross phenotype (body weight, BW 12.5 g) and unchanged blood ionized calcium concentration (1.30 mM) as compared to VDR^Δ/Δ^ single mutants (means ± s.d.; BW 13.3 ± 2.1 g, Ca^2+^ 1.24 ± 0.05 mM, *n* = 3), as well as *Fgf23*^−/−^/VDR^Δ/Δ^ (BW 12.5 ± 1.3 g, Ca^2+^1.24 ± 0.04 mM, *n* = 3) and *Kl*^−/−^/VDR^Δ/Δ^ (BW 12.1 ± 0.5 g, Ca^2+^ 1.20 ± 0.13 mM, *n* = 3) double mutant littermates (O. Andrukhova *et al*, unpublished data). Although obviously still preliminary, these data support the hypothesis that Klotho does not have VDR-and FGF23-independent actions on calcium metabolism.

In agreement with our finding that protein expression of TRPV5 did not differ between kidneys of WT and VDR^Δ/Δ^ mice, earlier studies reported unchanged renal TRPV5 mRNA expression in VDR-ablated mice (Weber *et al*, 2001; Okano *et al*, 2004). However, administration of pharmacological 1,25(OH)_2_D doses to mice or stimulation of primary cultures of murine renal tubular cells with pharmacological doses of 1,25(OH)_2_D directly increases transcription of the TRPV5 gene in a VDR-dependent fashion (Okano *et al*, 2004). Taken together, these data suggest that vitamin D is a pharmacological, but not a physiological regulator of TRPV5 protein expression in the murine kidney. Along similar lines, some previous experimental evidence suggested that vitamin D signaling may upregulate renal Klotho mRNA expression (Tsujikawa *et al*, 2003; Li *et al*, 2011). However, our data clearly showed that neither absent VDR signaling nor pharmacological stimulation with rFGF23 alters renal Klotho protein expression, suggesting only little physiological regulation by vitamin D and FGF23 at the level of the co-receptor *in vivo*.

It is well known that FGF23 is primarily a phosphaturic hormone, suppressing transcellular phosphate transport in proximal tubules. We previously reported that Klotho is expressed in the basolateral membrane of proximal tubular epithelium, and that FGF23 directly acts on proximal tubular cells to down-regulate membrane abundance of NaPi-2a through the ERK1/2-SGK1 – Na^+^/H^+^ exchange regulatory cofactor (NHERF)-1 signaling axis (Andrukhova *et al*, 2012). In contrast, Farrow and coworkers (Farrow *et al*, 2009) found that the earliest changes in activation of ERK1/2 after injection of FGF23 *in vivo* in mice occur in the distal tubules. It was previously thought that FGF23 acts on the distal tubule where it generates an unknown endocrine or paracrine secondary signal that in turn signals back to the proximal tubule to downregulate transcellular phosphate transport as part of a “distal-to-proximal tubular feedback mechanism” (White & Econs, 2008; Farrow *et al*, 2010; Martin *et al*, 2012). Our current study is able to explain this apparent discrepancy: Circulating FGF23 has parallel and independent, direct effects on proximal and distal renal tubules, suppressing phosphate re-uptake in proximal and stimulating calcium reabsorption in distal tubular cells. In both proximal (Andrukhova *et al*, 2012) and distal tubular epithelium, ERK1/2 and SGK1 activation are the initial downstream signaling events after binding of FGF23 to the binary FGFR-Klotho complex. Based on our *in vitro* experiments in isolated distal tubular segments, FGF23-induced activation of the PI3K pathway may also play an additional role in the regulation of transepithelial ion transport, at least in distal tubules.

Earlier studies showed that PTH increases transcription (Okano *et al*, 2004), and activates membrane-anchored TRPV5 by protein kinase A-mediated phosphorylation (de Groot *et al*, 2009). Thus, it is interesting to note that in both proximal and distal renal tubules, FGF23 and PTH signaling converge on the same molecules. In the proximal tubule, we and others showed that PTH and FGF23 signaling converge on NHERF-1 (Weinman *et al*, 2011; Andrukhova *et al*, 2012) to downregulate NaPi-2a membrane abundance, whereas in the distal tubule both signaling pathways converge on TRPV5. In the model shown in Fig 1B, PTH increases the open probability of membrane-anchored TRPV5 by protein kinase A-mediated phosphorylation, whereas FGF23 signaling is required for membrane transport of the channel. Although at present *in vivo* data supporting this hypothesis are lacking, our model would predict that the levels of circulating FGF23 may modulate renal sensitivity to the stimulating action of PTH on renal calcium re-absorption.

Our study has established a novel link between FGF23 signaling and WNK4 activation in distal tubular epithelium. FGF23 signaling resulted in increased phosphorylation of WNK4. The immunoelectron microscopic analysis clearly showed that FGF23 induces intracellular redistribution of WNK4 to the area beneath the apical cell membrane, where membrane vesicles fuse with the outer cell membrane. Thus, the most important function of WNK4 in the regulation of TRPV5 membrane abundance in the DCT *in vivo* may be the control of membrane vesicle transport and facilitation of vesicle fusion with the apical membrane, rather than the control of clathrin-dependent endocytosis as suggested by some *in vitro* experiments (Cha & Huang, 2010). The function of WNK4 at focal sites in the basal labyrinth as well as the function of TRPV5 expressed in the basal labyrinth of distal tubular cells remains unknown. Because WNK4 is involved in membrane transport of other ion channels such as NCC and ROMK1, FGF23 signaling may also influence the membrane abundance of other ion channels in distal tubules. In this context, it is interesting to note that *Kl* hypomorphic mice have decreased sodium plasma levels and increased circulating aldosterone concentrations (Fischer *et al*, 2010), suggesting that *Klotho* deficiency, and thus reduced FGF23 signaling, may induce alterations in renal sodium handling. WNK kinases act as a complex of at least 3 different kinases, WNK1, 3, and 4, to control the intracellular transport of membrane proteins (McCormick *et al*, 2008). In the current report, we focused on WNK4, but it is possible that FGF23 signaling involves other members of the WNK family as well. Clearly, more experimentation is required to elucidate all ramifications of the FGF23 signaling pathway in renal tubular epithelium.

Our finding that FGF23 stimulates renal tubular calcium reabsorption is important for our understanding of the endocrine networks involved in calcium and phosphate homeostasis, and suggests that in physiological situations where phosphate excretion needs to be increased extracellular calcium is still conserved. The pathophysiological downside of this regulation may occur in diseases such as chronic kidney disease, where PTH and FGF23 are chronically elevated due to decreased renal 1,25(OH)_2_D production and phosphate retention. In this situation, both hormones will stimulate renal calcium conservation, possibly contributing to calcium accumulation and vascular calcifications in these hyperphosphatemic patients.

## Materials and Methods

### Animals

All animal procedures were approved by the Ethical Committee of the University of Veterinary Medicine Vienna. Heterozygous VDR^+/Δ^ (Erben *et al*, 2002) were mated with heterozygous *Fgf23*^*+/−*^ (Sitara *et al*, 2004), and heterozygous *Klotho*^*+/−*^ (Lexicon Genetics, Mutant Mouse Regional Resource Centers, University of California, Davis, CA, USA) mutant mice to generate double heterozygous animals. VDR^+/Δ^/*Fgf23*^+/*−*^ and VDR^+/Δ^/*Klotho*^+/*−*^ mutant mice on C57BL/6 background were interbred to generate WT, VDR^Δ/Δ^, *Klotho*^*−*/*−*^, *Fgf23*^*−*/*−*^ and compound VDR^Δ/Δ^/*Fgf23*^*−*/*−*^ and VDR^Δ/Δ^/*Klotho*^*−*/*−*^ mutant mice. Genotyping of the mice was performed by multiplex PCR using genomic DNA extracted from tail as described (Hesse *et al*, 2007; Anour *et al*, 2012). The mice were kept at 24°C with a 12 h/12 h light/dark cycle, and were allowed free access to a rescue diet and tap water. The rescue diet (Ssniff, Soest, Germany) containing 2.0% calcium, 1.25% phosphorus, 20% lactose and 600 IU vitamin D/kg was fed starting from 16 days of age. This diet has been shown to normalize mineral homeostasis in VDR-ablated mice (Li *et al*, 1998; Erben *et al*, 2002; Zeitz *et al*, 2003). All experiments were performed on male offspring of double heterozygous × double heterozygous matings. Urine was collected in metabolic cages before necropsy. Some mice received a single intraperitoneal injection of vehicle (phosphate-buffered saline (PBS) with 2% DMSO) or recombinant human FGF23 R176/179Q (rFGF23) (Goetz *et al*, 2007) (10 μg per 3–4-month-old mouse; 5 μg per 9-month-old mouse) or), and were killed 8 or 24 h post-injection. In the mice receiving rFGF23, spontaneous urine was collected before as well as 8 and 24 h post-injection. At necropsy, the mice were exsanguinated from the abdominal V. cava under anesthesia with ketamine/xylazine (67/7 mg/kg i.p.) for serum collection. In all experiments 4–9 mice were used per experimental group.

### Serum and urine biochemistry

Serum creatinine, calcium, and phosphorus as well as urinary creatinine, calcium, and phosphorus were analyzed on a Hitachi 912 Autoanalyzer (Boehringer Mannheim) or on a Cobas c 111 analyzer (Roche). Serum Klotho protein concentrations were determined using a mouse specific Klotho ELISA kit (Cusabio) according to the manufacturer's protocol. The detection limit of the latter assay is 0.8 pg/ml.

### Immunohistochemistry

For immunohistochemistry, 5-μm-thick paraffin sections of paraformaldehyde (PFA)-fixed kidneys were prepared. Some sections were stained with hematoxylin/eosin (H & E) by routine methods. Before immunofluorescence staining, dewaxed sections were pretreated for 60 min with blocking solution containing 5% normal goat serum in PBS with 0.1% bovine serum albumin and 0.3% Triton X-100. Without rinsing, sections were incubated with polyclonal rabbit anti-αKlotho (Alpha Diagnostics, 1:1,000; raised against the cytoplasmic region of αKlotho, detecting the membrane-bound form), polyclonal rabbit anti-αKlotho (abcam, 1:1,000; raised against a part of KL2, detecting membrane-bound and ectodomain shed forms), anti-WNK4 (Novus Biologicals, 1:300), anti-TRPV5 (Alpha Diagnostics, 1:1,000), anti-β-actin (Sigma, 1:5,000), or anti-calbindin D-28k (Swant, 1:1,000) antibodies at 4°C overnight. After washing, sections were incubated for 1.5 h with goat anti-rabbit Alexa 548 and goat anti-mouse Alexa 488 secondary antibodies (Invitrogen, 1:400), respectively. Immunostaining of tissue sections in which either one or both secondary antibodies were omitted served as a negative control. The slides were analyzed on a Zeiss LSM 510 Axioplan 2 confocal microscope equipped with a 63 × oil immersion lens (NA 1.3). By use of the multitrack function, individual fluorochromes were scanned with laser excitation at 488 and 543 nm separately with appropriate filter sets to avoid cross talk. Pictures were processed using Adobe Photoshop (overlays). Quantification of distal tubular TRPV5 and WNK4 co-localization was performed using ImageJ software (National Institutes of Health, Bethesda, MA, USA) in four animals per group with 3–6 images per animal and 4–6 different regions of interest per image.

### Immuno-electron microscopic analysis

For transmission electron microscopy, kidney samples of 4 animals per group were fixed in 4% PFA for 24 h at 4°C. Longitudinal 100-μm-thick sections were cut using a Leica VT1000 Vibratome (Leica Microsystems). Sections were post-fixed in 4% PFA for 1 h and rinsed in 0.1 M phosphate buffer 3 × 10 min. Peroxidase activity was inhibited by 30-min incubation with 3% H_2_O_2_ in PBS for and nonspecific binding was minimized by incubation for 60 min in 3% normal goat serum containing 1% BSA. Incubation with anti-TRPV5 (Alpha Diagnostics, 1:150), anti-WNK4 (Novus Biologicals, 1:500), anti-αKlotho (Alpha Diagnostics, 1:300), anti-αKlotho (abcam, 1:400) or anti-calbindin D-28k (Swant, 1:500) was carried out at 4°C overnight. For negative controls, the primary antibody was omitted. Peroxidase-labeled rabbit PowerVision™ (ImmunoVision Technologies) secondary system was employed for antibody detection with subsequent diaminobenzidine (DAB, Sigma) staining. Post-fixation was performed in 1% osmium tetroxide for 2 h at RT followed by dehydration and incubation in propylene oxide, propylene oxide–epon and subsequent embedding in pure epon 812. Thin sections were stained with lead citrate, and were investigated under a transmission electron microscope (Zeiss EM 900). Apical expression of TRPV5 was quantified using Image J software by gray value determination in 4–5 images of four animals per group with 4–6 regions of interest in each image.

### Total cell membrane isolation

Mouse kidney cortex tissue was homogenized in a buffer consisting of 20 mM Tris (pH 7.4/HCl), 5 mM MgCl_2_, 5 mM NaH_2_PO_4_, 1 mM ethylenediamine tetraacetic acid (pH 8.0/NaOH), 80 mM sucrose, 1 mM phenyl-methylsulfonyl fluoride, 10 μg/ml leupeptin and 10 μg/ml pepstatin, and subsequently centrifuged for 15 min at 4,000 *g*. Supernatants were transferred to a new tube and centrifuged for an additional 30 min at 16,000 *g*.

### Isolation of distal tubular segments

Renal distal tubules were isolated as reported previously (Burg *et al*, 1997; Schafer *et al*, 1997; Gonzalez-Mariscal *et al*, 2000). In brief, murine kidneys were perfused with sterile culture medium (Ham's F12; GIBCO) containing 1 mg/ml collagenase (type II; Sigma) and 1 mg/ml pronase E (type XXV, Sigma) at pH 7.4 and 37°C. The cortical tissue was dissected in small pieces and placed at 37°C in sterile Ham's F12 medium containing 0.5 mg/ml collagenase II and 0.5 mg/ml pronase E for 15 min with vigorous shaking. After centrifugation at 3000 rpm for 4 min, the enzyme-containing solution was removed, and tubules were resuspended in ice-cold medium. Individual distal tubule segments were identified based on morphology in a dissection microscope at x25–40 magnification by their appearance and dimensions. Distal tubular segments were incubated with rFGF23 (100 ng/ml) and/or 10 ng/ml of the specific SGK1 kinase inhibitor GSK 650394 (Axon Medchem), or 10 ng/ml of the ERK1/2 inhibitor PD184352 (Sigma) for 1, 2 and 4 h. For experiments with FGFR inhibition, distal tubular segments were incubated with rFGF23 (100 ng/ml) and/or 40 ng/ml of the specific FGFR inhibitor PD173074 (Sigma) for 2 h. For experiments with SRC, PLC and PI3K inhibition, distal tubular segments were incubated with rFGF23 (100 ng/ml) and/or 150 nM Scr inhibitor-1, 50 nM PI3K inhibitor wortmannin or 10 μM PLC inhibitor U-73122 (all from Sigma) for 2 h. Protein samples were collected for Western blotting analysis in lysis buffer.

### Western Blot

Kidney cortex homogenate, total cell membrane samples or dissected distal tubular segments were solubilized in Laemmli sample buffer, fractionated on SDS-PAGE (30 μg/well) and transferred to a nitrocellulose membrane (Thermo Scientific). For detection of Klotho protein in urine, 40 μl of fresh bladder urine, salt precipitated urine, or 1.3-and 2-fold concentrated urine using Amicon Ultra-0.5 centrifugal filters (Millipore) were loaded per well as described (Hu *et al*, 2010). Immunoblots were incubated overnight at 4°C with primary antibodies including polyclonal mouse anti-TRPV5 (1:3,000, Alpha Diagnostics), polyclonal rabbit anti-αKlotho (1:2,000, Alpha Diagnostics, membrane-bound form), polyclonal rabbit anti-αKlotho (1:2500, abcam; membrane-bound and shed forms), polyclonal rabbit anti-WNK4 (1:2,000, Novus Biologicals), polyclonal anti-calbindin D-28k (1:2,000, Swant), polyclonal anti-uromodulin (1:1,500, Sigma), monoclonal mouse anti-β-actin (1:5,000, Sigma), monoclonal anti-total-ERK1/2 (BD Biosciences), anti-phospho-ERK1/2 (Cell Signaling), anti-total-SGK1 (Alpha Diagnostics), and anti-phospho-SGK1 (Santa Cruz Biotechnology) in 2% (w/v) bovine serum albumin (BSA, Sigma) in a TBS-T buffer [150 mM NaCl, 10 mM Tris (pH 7.4/HCl), 0.2% (v/v) Tween-20]. After washing, membranes were incubated with horseradish peroxidase-conjugated secondary antibodies (Amersham Life Sciences). Specific signal was visualized by ECL kit (Amersham Life Sciences). The protein bands were quantified by Image Quant 5.0 software (Molecular Dynamics). The expression levels were normalized to Ponceau S stain and to individual β-actin expression levels. The glycosylation of the Klotho and TRPV5 proteins was detected on nitrocellulose membranes by GLYCO-PRO assay (Sigma) according to the manufacturer's protocol. For validation of anti-TRPV5 antibody specificity, total kidney homogenates of 4-week-old TRPV5^−/−^ male mice (generous gift of René J.M. Bindels, University Medical Center Nijmegen, Netherlands) were used as a negative control. Each experiment included 4–8 animals per group. All presented Western blot images are from the same gel each. All splicing events are indicated by frames in Western blot images.

### Co-immunoprecipitation

Kidney cortex homogenate protein samples (1 mg), dissected proximal tubular segments (40 μg), or homogenate protein samples from murine smooth muscle cells (1 mg) were incubated with 2 μg of WNK4 (Novus Biologicals), TRPV5 (Lifespan Biosciences), anti-phosphoserine (Alpha Diagnostics), or anti-NaPi-2c (kind gift of Drs. Murer and Biber) antibody at 4°C overnight. The immune complexes were captured by adding 50 μl Protein A or G agarose/sepharose beads (Santa Cruz Biotechnology) and overnight incubation at 4°C with gentle rocking. The immunoprecipitates were collected by centrifugation at 1,000 *g* for 5 min at 4°C and washed for four times in PBS, each time repeating the centrifugation step. After the final wash, the pellets were suspended in 40 μl of electrophoresis sample buffer and boiled for 2–3 min. Western blot analysis was performed using a primary anti-TRPV5 or anti-WNK4 antibody. Each experiment was performed in duplicates and included 4–8 animals per group.

### RNA isolation and quantitative RT-PCR

Shock-frozen tissues were homogenized in TRI Reagent (Molecular Research Center) and total RNA was extracted according to the manufacturer's protocol. RNA purity and quality was determined using a 2100 Bioanalyzer (Agilent Technologies). One μg of RNA was used for first-strand cDNA synthesis (iScript cDNA Synthesis Kit, Bio-Rad). Quantitative RT-PCR was performed on a Rotor-Gene™ 6000 (Corbett Life Science) using QuantiFast™ EverGreen PCR Kit (Qiagen). A melting curve analysis was done for all assays. Primer sequences are available on request. Efficiencies were examined based on a standard curve. Expression of target genes was normalized to the expression of the housekeeping gene glyceraldehyde-3-phosphate-dehydrogenase (GAPDH).

### *In vitro* transient transfection experiments

The pCMV6 plasmid construct containing mouse TRPV5 ORF (MR225560, Origene), mouse SGK1 and mouse WNK4 (generous gifts of David H. Ellison, Oregon Health and Science University, Portland, Oregon, USA) were used for *in vitro* transient transfection experiments. MDCK (Sigma) cells were grown in EMEM (EBSS) with 2 mM glutamine, 1% non-essential amino acids, 10% fetal bovine serum and 100 μg/ml penicillin and 100 μg/ml streptomycin at 37°C in a 5% CO_2_/95% air humidified atmosphere. 24 h after seeding, cells at ˜70% confluence were subjected to transient transfection using X-tremeGENE 9DNA transfection reagent (Roche) according to the manufacturer's protocol. The cells were co-transfected with 0.5 μg of each construct. 24 h post-transfection, the cells were treated with either vehicle or 100 ng/ml rFGF23 and/or 10 ng/ml recombinant mouse Klotho (R&D Systems). Complex glycosylated TRPV5 expression was monitored by Western blotting in triplicate 12 h after cell stimulation.

### Ca^2+^ imaging and image analysis

The MDCK cells and kidney slices (300-μm-thick) were incubated for 30 min at 37°C with 2 μM of the calcium-sensitive dye Fluo-4 (Molecular Probes), diluted in DMSO (Merck Millipore International) and 20% Pluronic (Merck Millipore International). Thereafter, the slices or cells were washed two times for 15 min each in PBS. Some kidney slices from WT mice were incubated *in vitro* for 2 h with rFGF23 (100 ng/ml) or vehicle. For calcium visualization in kidney slices, Fluo-4 was excited by a two-photon laser (Ti-sapphire laser, Coherent Inc.) at a wavelength of 820 nm. Images (512 × 512 pixels) were acquired every 30 s at a depth of 50–80 μm. For inhibition of TRPV activity, tissue slices were incubated *ex vivo* with 1–50 μM of ruthenium red (Sigma) at 37°C, 5% CO_2_/95% air humidified atmosphere for 30 min. Fluorescence images were analyzed using Image J software. The whole epithelial layer of the tubule except for the lumen was selected (by manually drawing the region of interest, ROI) to quantify Ca^2+^ levels in renal distal tubules. Fluorescence intensity was quantified in 7–9 ROIs per experimental group from three independent experiments. The ratio between the fluorescence intensity and the ROI was calculated for each tubule.

Fluorescence imaging of cultured cells was performed using an inverse confocal microscope (TCS SP5 Leica Microsystems), with a Leica water immersion objective (63×). Fluo-4 was excited by an argon laser at 488 nm. Images were acquired every 30 s. Ruthenium red (1–10 μM) was used as TRPV inhibitor. In some experiments, 1.5 mM EGTA (Sigma) was added to the culture medium to chelate extracellular calcium. For each MDCK cell, the whole-cell and nucleus contours were drawn manually. The mean fluorescence intensity was measured in 4–8 separate cells from each field of view (2–7 fields of view per individual sample). The ratio of the fluorescence intensity and the cell square was calculated for each cell (16–48 cells per individual sample).

### Statistical analyses

Statistics were computed using PASW Statistics 17.0 (SPSS Inc., Chicago, IL, USA) The data were analyzed by two-sided *t*-test (2 groups) or 1-way analysis of variance (ANOVA) followed by Student-Newman-Keuls multiple comparison test (>2 groups). *P* values of less than 0.05 were considered significant. The data are presented as the mean ± s.e.m.
