# Supplementary material for: FGF23 promotes renal calcium reabsorption through the TRPV5 channel
Source: EMBO J. 2014 Jan 17;33(3):229–46. doi: 10.1002/embj.201284188 (PMC3983685; doi:10.1002/embj.201284188)
Supplement: Supplementary file 1 [file embj0033-0229-sd1.pdf]

Supplementary Figure legends

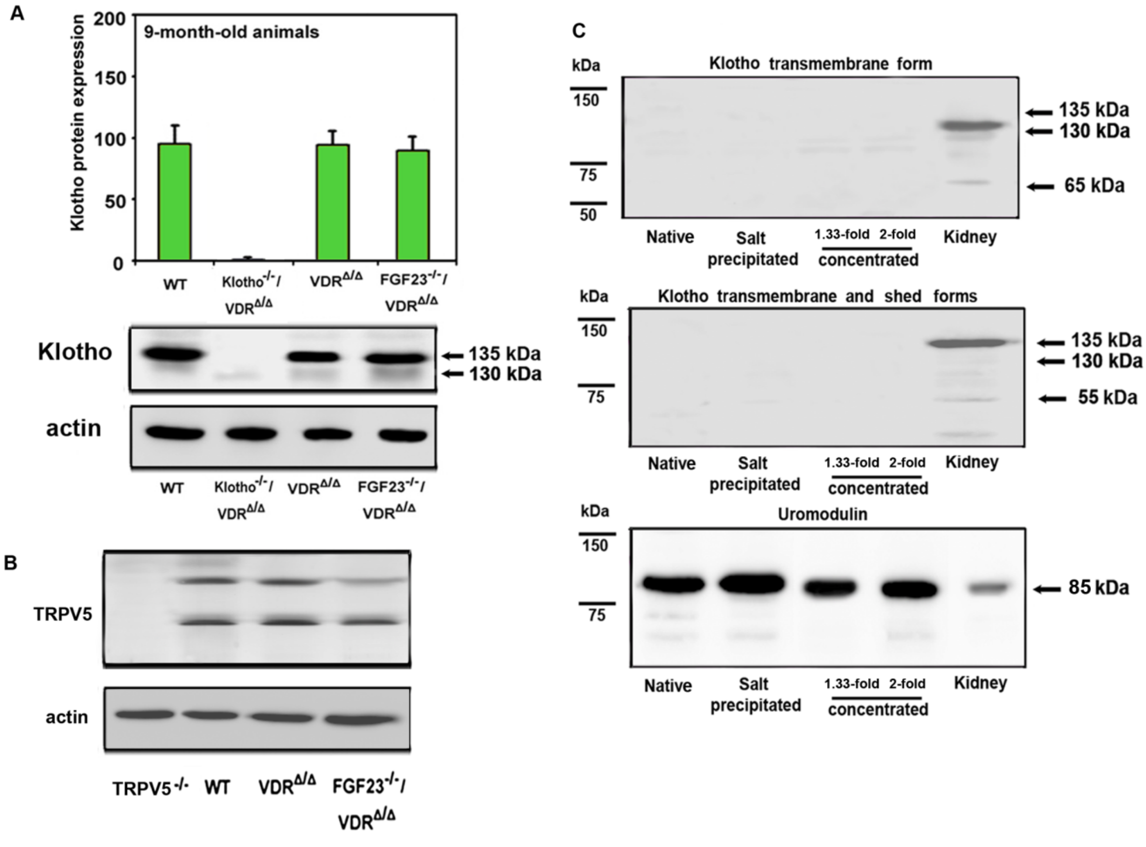

Figure S1. Renal αKlotho protein abundance is unchanged in *Fgf23* and *Klotho* deficient mouse models and αKlotho is undetectable in urine of wild-type mice. A. Western blot analysis of αKlotho in renal total protein extracts in 9-month-old male WT, $VDR^{\Delta/\Delta}$, $Kl^{-/-}$/$VDR^{\Delta/\Delta}$, and $Fgf23^{-/-}$/$VDR^{\Delta/\Delta}$ mice on rescue diet, using an anti-Klotho antibody detecting the membrane-bound and the ectodomain shed form of the protein. Only the 135 kD transmembrane isoform of Klotho was quantified. Data represent mean ± SEM of 3 to 4 animals each. **B.** Specificity of the anti-TRPV5 antibody was controlled by Western blot analysis of renal total protein extracts from $TRPV5^{-/-}$, WT, $VDR^{\Delta/\Delta}$ and $Fgf23^{-/-}$/$VDR^{\Delta/\Delta}$ mutants (n=3-6). **C.** Western blot analysis of αKlotho and uromodulin in native, salt precipitated and concentrated (1.33- and 2-fold) urine samples from WT mice (n=5-6) . Renal total protein extract from WT mouse and anti-uromodulin antibody were used as positive controls. Anti-Klotho antibodies detecting the membrane-bound (anti-cytoplasmic domain, upper panel) or the membrane-bound and ectodomain shed (anti KL2 domain, lower panel) forms of the protein were used.
