# Supplementary material for: FGF23 promotes renal calcium reabsorption through the TRPV5 channel
Source: EMBO J. 2014 Jan 17;33(3):229–46. doi: 10.1002/embj.201284188 (PMC3983685; doi:10.1002/embj.201284188)
Supplement: Supplementary file 2 [file embj0033-0229-sd2.pdf]

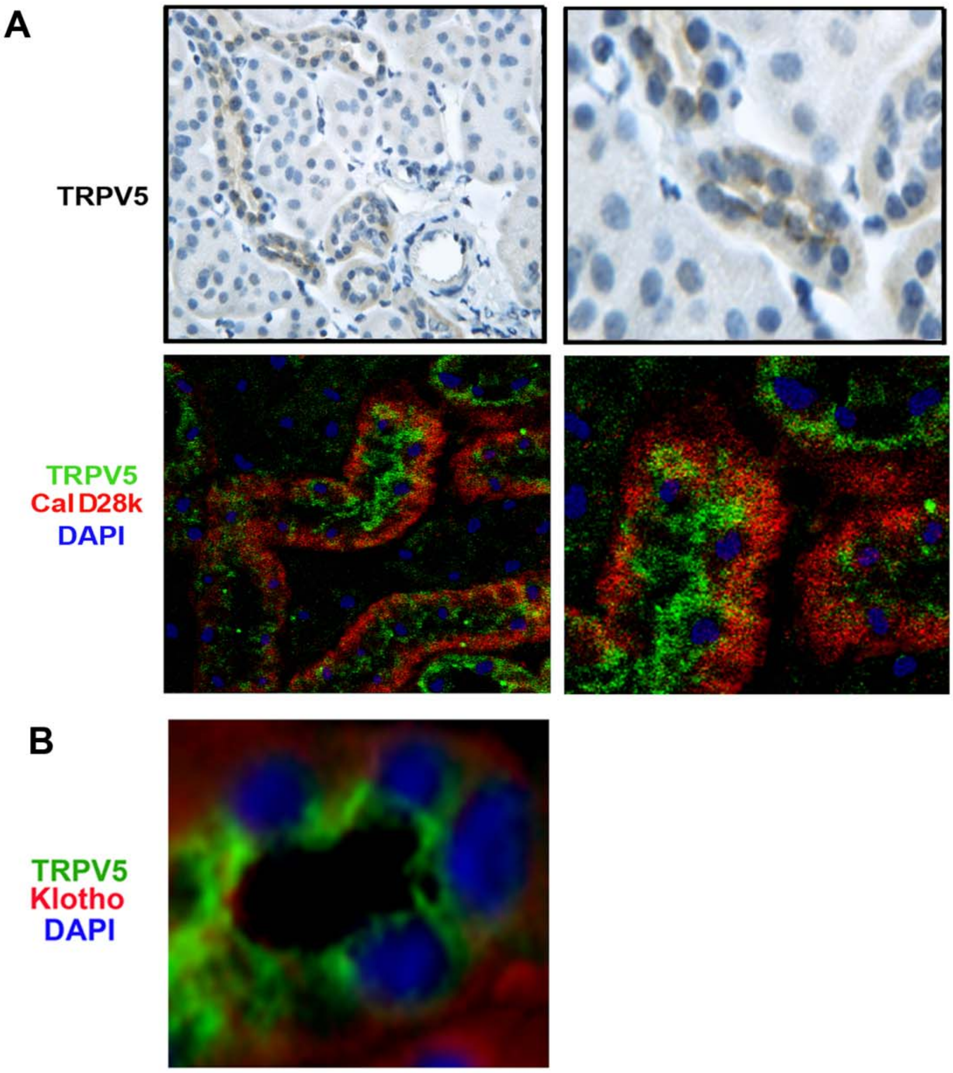

Supplemental Figure 2, Andrukhova et al.

Figure S2. TRPV5 and calbindin D28k are exclusively expressed in renal distal renal tubules, but TRPV5 does not co-localize with membrane-bound or ectodomain shed αKlotho. A. Light microscopic immunohistochemical staining with anti-TRPV5 (upper panels, brown-stained DAB is used for detection) or immunofluorescence co-staining (lower panels) with anti-TRPV5 (green), anti-calbindin D28k (red), and DAPI (blue) of paraffin sections from kidneys of 4-month-old WT mice (n=5-7). TRPV5 staining is observed exclusively in distal tubules co-expressing calbindin D28k (CalD-28k). Right panels show higher magnification. **B.** Immunohistochemical co-staining with anti-αKlotho (red) antibody raised against the KL2 domain (detecting membrane-bound and ectodomain shed form of the protein), anti-TRPV5 (green), and DAPI (blue) of paraffin sections from kidneys of 4-month-old WT mouse (n=5). Original magnification x630.
