# Supplementary material for: FGF23 promotes renal calcium reabsorption through the TRPV5 channel
Source: EMBO J. 2014 Jan 17;33(3):229–46. doi: 10.1002/embj.201284188 (PMC3983685; doi:10.1002/embj.201284188)
Supplement: Supplementary file 3 [file embj0033-0229-sd3.pdf]

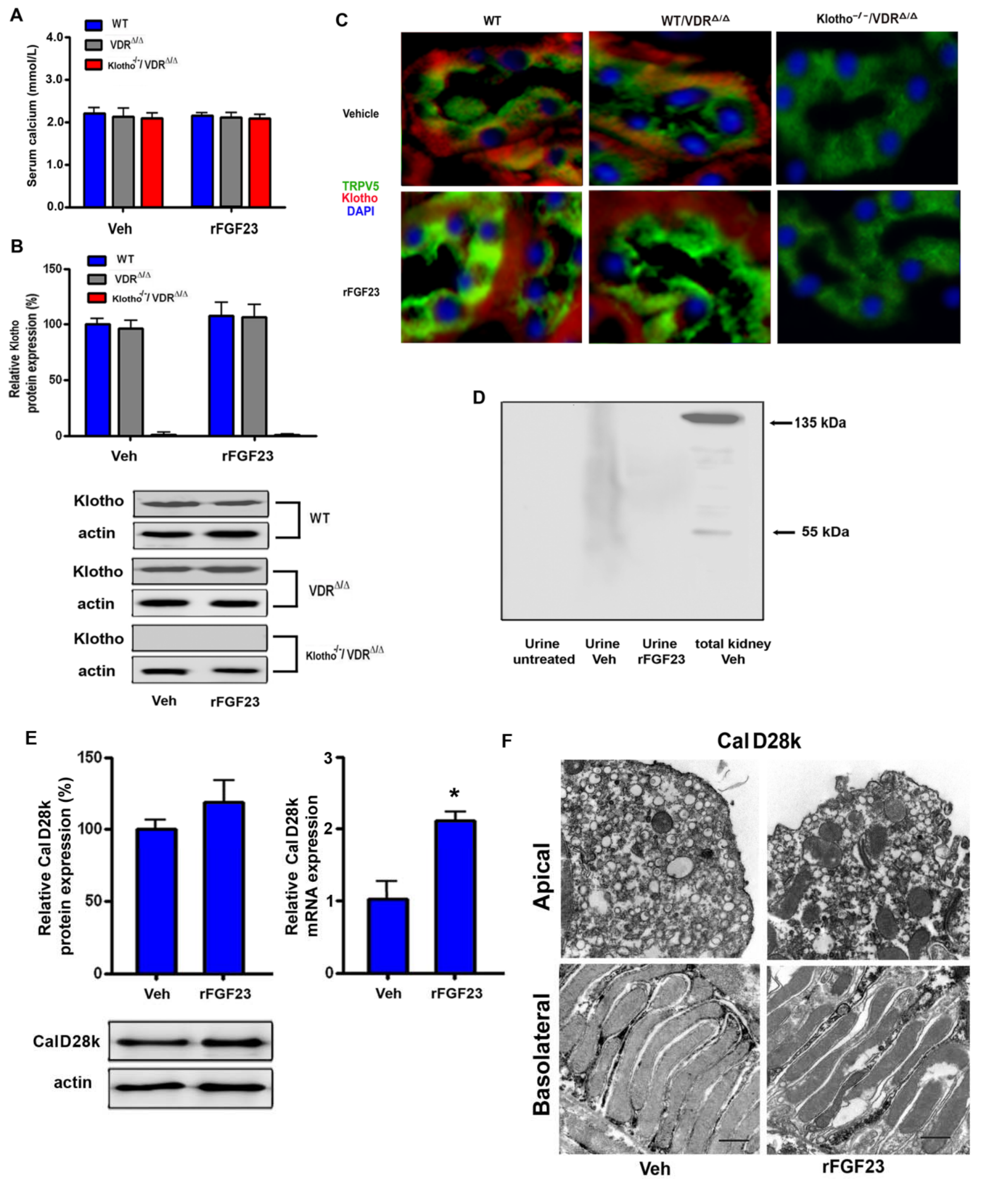

Supplemental Figure 3, Andrukhova et al.

Figure S3. FGF23 treatment of wild-type mice has no effect on renal αKlotho protein expression and subcellular distribution of calbindin D28k. A. Serum calcium in 4-month-old WT mice injected i.p. with vehicle or a single, 10 µg dose of rFGF23, 24 hours post-injection. Data represent mean ± SEM of 3 to 5 animals each. **B.** Western blot analysis of αKlotho (antibody detecting membrane-bound and shed forms) in renal total protein extracts from 4-month-old WT, VDR$^{\Delta/\Delta}$, and *Kl*$^{-/-}$/VDR$^{\Delta/\Delta}$ mice treated with vehicle or rFGF23 (10 µg/mouse) 8 hours before necropsy. Data represent mean ± SEM of 3 – 5 animals each. **C.**

Immunohistochemical co-staining with anti-αKlotho (red) antibody raised against the KL2 domain (detecting membrane-bound and ectodomain shed form of the protein), anti-TRPV5 (green), and DAPI (blue) of paraffin sections from kidneys of 4-month-old WT, $VDR^{\Delta/\Delta}$, and $Kl^{-/-}/VDR^{\Delta/\Delta}$ mice on rescue diet (n=3-6). Original magnification x630. **D.** Western blot analysis of αKlotho (antibody detecting membrane-bound and shed forms) in urine from 4-month-old WT mice with no treatment or 8 hours after injection of either vehicle or 10 µg of rFGF23 (n=5). Renal total protein extract from WT mice was used as a positive control. **E.** Protein and mRNA abundance of calbindin D28k in renal total extracts of 4-month-old WT mice treated with vehicle or 10 µg of rFGF23, 8 hours post-injection. Data represent mean ± SEM of 5 animals each. **F.** Immuno-electron microscopic staining using anti-calbindin D28k antibodies in kidneys from 4-month-old WT mice treated with vehicle (Veh) or 10 µg of rFGF23, 8 hours before necropsy (n=5). Upper panels show apical areas, lower panels show basolateral areas of distal tubular cells. Bar = 500 nm.
