# Supplementary material for: FGF23 promotes renal calcium reabsorption through the TRPV5 channel
Source: EMBO J. 2014 Jan 17;33(3):229–46. doi: 10.1002/embj.201284188 (PMC3983685; doi:10.1002/embj.201284188)
Supplement: Supplementary file 4 [file embj0033-0229-sd4.pdf]

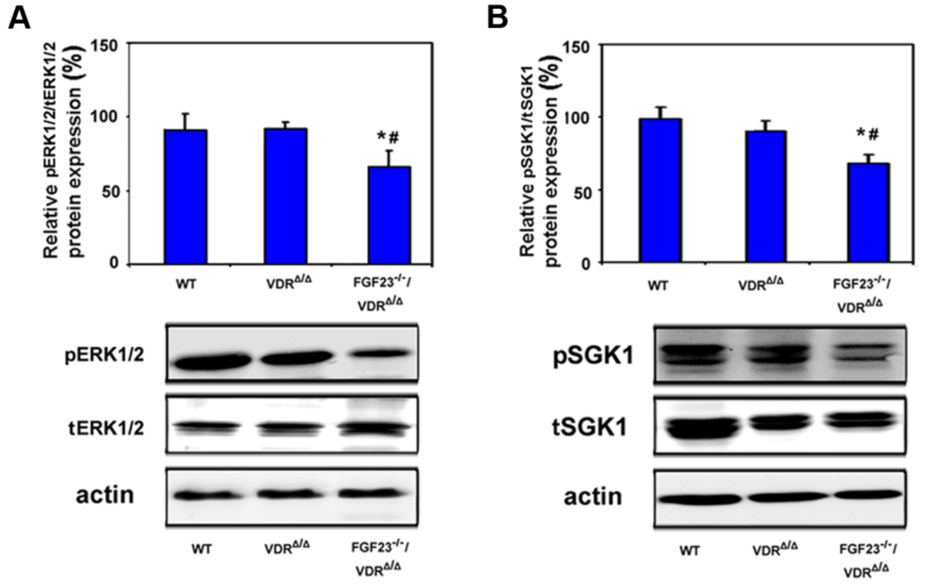

Supplemental Figure 4, Andrukhova et al.

Figure S4. ERK1/2 and SGK1 phosphorylation is decreased in *Fgf23* deficient mice. (**A**) Western blot analysis of phosphorylated ERK1/2 and (**B**) phosphorylated SGK1 in homogenized renal cortex samples of 9-month-old WT, $VDR^{\Delta/\Delta}$ and $Fgf23^{-/-}/VDR^{\Delta/\Delta}$ mice. Data represent mean ± SEM of 6 – 8 animals each. * denotes $P < 0.05$ vs. WT, # $P < 0.05$ vs. $VDR^{\Delta/\Delta}$ mice.
