# Supplementary material for: FGF23 promotes renal calcium reabsorption through the TRPV5 channel
Source: EMBO J. 2014 Jan 17;33(3):229–46. doi: 10.1002/embj.201284188 (PMC3983685; doi:10.1002/embj.201284188)
Supplement: Supplementary file 5 [file embj0033-0229-sd5.pdf]

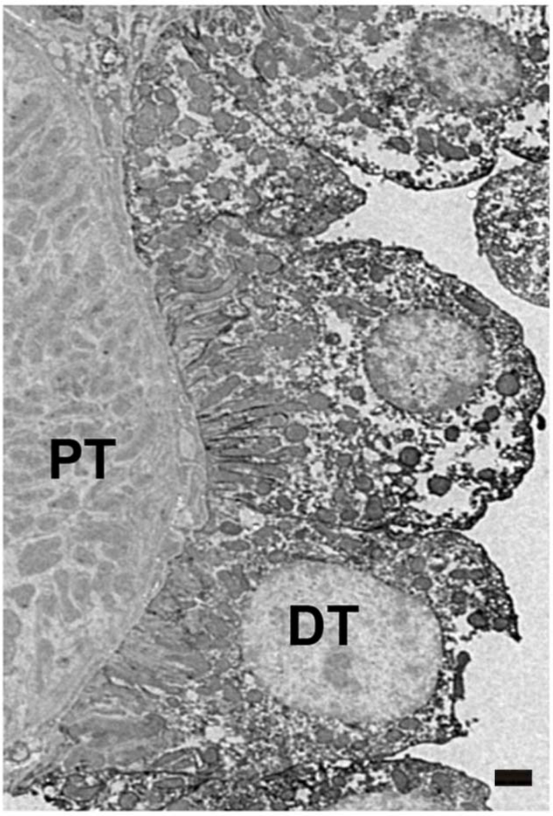

Supplemental Figure 5, Andrukhova et al.

Figure S5. Anti-TRPV5 antibody stains exclusively renal distal tubular cells. Overview of immuno-electron microscopic staining using an anti-TRPV5 antibody, and DAB for detection of bound antibody. DAB staining (black) is found exclusively in distal tubular cells. PT = proximal tubular cell, DT = distal tubular cell. Bar = 500 nm.
