# Supplementary material for: FGF23 promotes renal calcium reabsorption through the TRPV5 channel
Source: EMBO J. 2014 Jan 17;33(3):229–46. doi: 10.1002/embj.201284188 (PMC3983685; doi:10.1002/embj.201284188)
Supplement: Supplementary file 6 [file embj0033-0229-sd6.pdf]

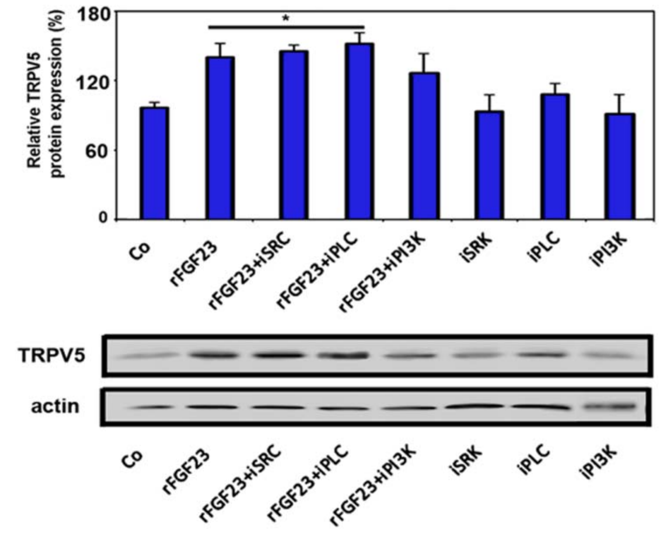

Supplemental Figure 6, Andrukhova et al.

Figure S6. FGF23-induced increase in TRPV5 protein abundance in isolated distal tubular segments is independent of SRC and PLC signaling. Complex glycosylated TRPV5 protein expression in isolated distal tubular segments treated *in vitro* with rFGF23 alone or in combination with specific inhibitors of Src kinase (iSRC), phospholipase C (PLC), or phosphatidylinositol 3-kinase (iPI3K). Data represent mean ± SEM of 3 – 4 individual samples. * denotes $P < 0.05$ vs. vehicle control (Co).
