# Supplementary material for: FGF23 promotes renal calcium reabsorption through the TRPV5 channel
Source: EMBO J. 2014 Jan 17;33(3):229–46. doi: 10.1002/embj.201284188 (PMC3983685; doi:10.1002/embj.201284188)
Supplement: Supplementary file 11 [file embj0033-0229-sd11.pdf]

Supplementary video legends

Supplementary video 1. Changes in fluorescence over time (30 min) after addition of 10 µM ruthenium red in distal tubules of a Fluo-4-loaded kidney slice from a 3-month-old rFGF23-treated mouse, 8 hours after injection.

Supplementary video 2. Changes in fluorescence over time (30 min) after addition of 10 µM ruthenium red in distal tubules of a Fluo-4-loaded kidney slice from a vehicle-treated mouse, 8 hours after injection.

Supplementary video 3. Time-dependent increase in intracellular fluorescence in distal tubules in a Fluo-4-loaded, 300-µm-thick kidney slice from of a 3-month-old wild-type mouse treated at time 0 with rFGF23 (100 ng/ml) *in vitro*.

Supplementary video 4. Decrease in intracellular fluorescence in distal tubules in a 300-µm-thick kidney slice from of 3-month-old wild-type mouse treated at time 0 with rFGF23 (100 ng/ml) *in vitro*. After 120, 135, and 150 min, 1, 10, and 50 µM of the TRPV inhibitor ruthenium red (RR) was added, respectively.
