# Supplementary material for: FGF23 promotes renal calcium reabsorption through the TRPV5 channel
Source: EMBO J. 2014 Jan 17;33(3):229–46. doi: 10.1002/embj.201284188 (PMC3983685; doi:10.1002/embj.201284188)
Supplement: Supplementary file 12 [file embj0033-0229-sd12.pdf]

THE EMBO JOURNAL

Manuscript EMBO-2012-84188

FGF23 Promotes Renal Calcium Reabsorption Through the TRPV5 Channel

Olena Andrukhova, Alina Smorodchenko, Monika Egerbacher, Carmen Streicher, Ute Zeitz, Regina Goetz, Victoria Shalhoub, Moosa Mohammadi, Elena E. Pohl, Beate Lanske, and Reinhold G. Erben

Corresponding author: Reinhold Erben, University of Veterinary Medicine Vienna

Review timeline:

Submission date:	10 December 2012
Editorial Decision:	07 February 2013
Revision received:	20 August 2013
Editorial Decision:	16 September 2013
Additional Author Correspondence:	20 September 2013
Additional Editorial Correspondence:	15 October 2013
Revision received:	30 October 2013
Accepted:	18 November 2013

Transaction Report:

(Note: With the exception of the correction of typographical or spelling errors that could be a source of ambiguity, letters and reports are not edited. The original formatting of letters and referee reports may not be reflected in this compilation.)

Editor: Anne Nielsen

1st Editorial Decision 07 February 2013

Thank you for submitting your manuscript for consideration by the EMBO Journal. It has now been seen by three referees whose comments are shown below. As you will see, while the referees all express interest in your findings, they also raise a number of critical concerns about the experiments provided to support the conclusions made in your manuscript.

It is particularly concerning that both referees #1 and #3 emphasize that additional control experiments are required to exclude the presence of secreted Klotho in mouse urine. Since the main message of your manuscript is to refute the previously proposed extracellular role for Klotho, this statement must be rigorously supported by the presented data. Along the same lines, you need to more directly assess TRPV5 channel activity as requested by ref#1 and to further dissect the functional causality in TRPV5 localization as a consequence of Klotho/Fgf23 signaling as outlined by ref#2.
Another important issue raised by all three referees is the need to substantially improve the quality, quantification and conclusiveness of the histological and immunohistochemical data provided in figs. 3, 4 and 6. Finally, while we will not make it an ultimate claim, we support the suggestion made by ref #3 to address the phenotype in Klotho-/-;Fgf23-/- mice, if these mice are available.

Should you be able to address all criticisms raised in the reports to the full satisfaction of all three referees, we could consider a revised manuscript. I should remind you, though, that it is EMBO Journal policy to allow a single round of revision only and that acceptance or rejection of the manuscript will therefore depend on the completeness of your responses in this revised version. I do realize that addressing all the referees' criticisms will require a lot of additional time and effort and be technically challenging. I would therefore understand if you wish to publish the manuscript rapidly and without any significant changes elsewhere, in which case please let us know so we can withdraw it from our system.

If you decide to thoroughly revise the manuscript for the EMBO Journal, please include a detailed point-by-point response to the referees' comments. Please bear in mind that this will form part of the Review Process File, and will therefore be available online to the community. For more details on our Transparent Editorial Process, please visit our website:
http://www.nature.com/emboj/about/process.html

We generally allow three months as standard revision time. As a matter of policy, competing manuscripts published during this period will not negatively impact on our assessment of the conceptual advance presented by your study. However, we request that you contact the editor as soon as possible upon publication of any related work, to discuss how to proceed. Should you foresee a problem in meeting this three-month deadline, please let us know in advance and we may be able to grant an extension.

Thank you for the opportunity to consider your work for publication.

REFEREE REPORTS:

Referee #1:

The manuscript of Andrukhova et al. describes a novel mechanism for the regulation of renal calcium reabsorption by Klotho and FGF23. The authors propose that FGF23 acts on a basolaterally located FGF23-receptor containing Klotho as a co-factor to increase apical membrane expression of the calcium entry channel TRPV5 via ERK`/2, SGK1 and WNK4. This mechanism is at odds with the mechanism proposed by Chang et al. (Science, 2005), who indicated that Klotho can be present on the apical side and in urine, and acts by hydrolizing extracellular sugar residues on the TRPV5 channel. Overall, the manuscript is interesting and of high quality.
Main points:

- It is not fully clear why some results are in discepancy with Chang et al. or whether both mechanisms could work in concert/parallel. The sentence on Page 7 "The fact that Klotho and Fgf23 deficiency have almost identical effects on renal TRPV5 is difficult to explain on the basis of the model shown in Fig. 1A" is meaningless, as Fig. 1A does not include Fgf23 or its receptor. The fact that Klotho deficiency and Fgf23 deficiency have the same effects does not invalidate the model in Fig. 1A. As outlined below, I don't see the real evidence in this paper that the model by Chang et al. is necessarily wrong; the mechanisms could work in concert/parallel.

- The only actual argument against the mechanism proposed by Chang et al. is that Andrukhova et al. fail to detect Klotho in urine (i.e. compare Fig. 2D with Fig. 1b in Chang et al.). There is no clear discussion why this difference could occur (differences in experimental protocol, mouse strain, antibody??), nor is there any positive control that the authors can detect any protein in WB on mouse urine. There are also other authors that found Klotho in urine using wb or ELISA. This point should be further discussed and positive controls of WB on urinary proteins should be included (e.g. uromodulin).

-The histological data (immunohistochemistry, immuno-electron microscopy) as shown are very much a "matter of belief". There is no quantification of crucial aspects such as colocalisation (e.g. Fig. 6A) or the degree of (sub)plasmalemmal localisation (e.g. Fig. 6C), nor is there an indication of the number of slides/animals that were analysed. Since these data are essential for the conclusions, some indication of the significance of the effects is essential.

-The paper lacks direct assesment of TRPV5 function. Although extreted calcium levels and membrane staining could well be indications of TRPV5 function, this is not necessarily the case. For example, PACSIN3 causes an increase in membrane expression of the related channel TRPV4, yet leads to a reduced channel function. To demonstrate that the proposed mechanism leads to increased TRPV5 channel function, some more direct measurements (e.g. patch-clamp, calcium uptake in TRPV5-expressing cells) need to be included.

Referee #2:

This reviewer supports the concept and conclusions described in this manuscript. The followings are commented to strengthen the concept and conclusions of authors.

Major points
1. The authors observed that urinal calcium excretion levels of alpha-Klotho (aKl)/VDR-double knockout-mice(KOM) and FGF23/VDR-double KOM are larger than that of VDR-single KOM. This fact possibly indicates that aKl/FGF23 signal makes a distinct effect rather than vitamin D suppression. Thus, the authors hypothesized that FGF23/aKl signaling pathway induces both TRPV5 expression and translocation to the apical lumen of the tubule. The reviewer has the following questions and comments.
A. The authors should show the general data of aKl/VDR-double KOM, FGF23/VDR-double KOM and VDR-single KOM, including Ca, Pi, PTH, VD3, calcitonin, FGF23, and bone mineral density. Based on severer impairment in Ca reabsorption, there may be different profile of mineral index between double- and single-KOM. Describe the details and discuss that point.
B. In general, urinary calcium reabsorption depends on a variety of cation channels in the tubular segments. The weakest point of the manuscript is that the authors did not address whether abundant urinary Ca excretion in double-KOM directly depends on TRPV5 translocation in the tubule cells. The authors should explain how they concluded TRPV5 as the cause of urinary Ca excretion.
2. The authors proposed that FGF23/aKlotho signaling induces TRPV5 translocation through pERK1/2, pSGK1, WNK4 kinase pathway. The pathway seems reasonable. To strengthen this hypothesis, it would be better that the authors reconstitute TRPV5 translocation system using the relevant signaling molecules in cultured cells such as MDCK.
3. The authors observed TRPV5 intracellular location of the tubular cells mainly using immuno-histochemistry (Figure3,4) and the histological images were used for the chief evidences to demonstrate aKl/FGF23-depedent TRPV5 translocation system. However, unclear images did not necessarily address the convincing conclusion. The authors have to obtain clearer images using rational control, such as increased calbindin D and NaPi2c translocation to the plasma membrane on vitamin D and FGF23 injection, respectively. EM images of Figure 6 also could not succeed in clarifying the inter-organellar transport (as described in page 11).

Minor point
1. In Figure 4 B, serum PTH of VDR-KOM is maintained at approximately 50pg/ml. Even fed with rescue diet, it seems curious that the PTH level is completely restored. In VDR-KO state, dehydration will be quietly enhanced for abundant Ca excretion. For example, mineral contents of supplied water need to be checked, if the authors did not choose deionized water. In addition, the authors are requested to explain the phenomenon that vehicle administration resulted in continuous PTH secretion.

Referee #3:

Previous studies based mostly on in vitro assays had proposed that klotho could have an effect on the activity of the TRPV5 channel by directly and physically regulating the glyosylation state of TRPV5 thanks to its putative glycosidase activity.
Andrukhova et al now use a series of animal models to elegantly study the cross-talks between klotho, the TRPV5 channel and regulation of Calcium homeostasis in the distal tubules of the kidney. The results provided suggest an alternative explanation for previous findings which appears more in line with the accumulating evidence of klotho acting mainly as a co-receptor for FGFR in response to FGF23 stimutaion.

© EMBO

The study is certainly worth of notice as it builds on a careful, systematic and accurate analysis of the system in physiological conditions which is likely to provide a credible mechanistic explanation for the cross-talk between Klotho and the TRPV5, which appears to be indirect via signaling of SGK1 and WNK4.

This reviewer has two main concerns with the data:
-The piece of data which appears to be key and clearly highlights the need for proposing the model shown in Figure 1B as opposed to the model proposed in Figure 1A is the subcellular localization of klotho. The authors use nicely controlled studies of Kl-/- tissues to characterize two distinct antibodies directed against klotho. The western blot analysis is convincing and shows specific immunodetection. On the basis of this specificity in western blot analysis the data showing lack of klotho in the urines (Supplementary Figure 3D) are very important. Could the authors attempt at performing protein enrichment in the urine such as salt precipitation or volume decrease to make sure that minimal amounts of klotho are not detectable in the urines?
The immunolocalization studies shown in Figure 3B do not appear very specific, as the staining appears to be rather diffused. Having in hands klotho-/- tissues, again, could the authors attempt at performing immuno-EM localization of klotho as a more precise evidence of its basal localization?

- Second, based on their studies the authors propose that klotho regulates the TRPV5 distribution and function indirectly, acting as a co-receptor for the FGFreceptor (on the basolateral side of cells, see above). The data showing similar pohenotypes in the Fgf23-/- and kl-/- animals (both on VDRdelta/delta background) indeed strongly suggest that the specific function of klotho on TRPV5 and calciuria are mediated via its classical function of co-receptor for FGF23. The obvious subsequent experiment that would definitively demonstrate this would be the generation of double mutant animals (triple in this case: Kl-/-;FGF23-/-;VDRdelta/delta) and the analysis of the phenotype which should be identical to the individual mutants (Kl-/- and FGF23-/-, respectively) if indeed their interaction is the the only mechanism acting here.

Minor:
-Both in figure 5D and 7D, the loading control is not povided. Immunoprecipitation of WNK4 followed by Immunoblotting with anti-P-ser shows increased phosphorylation of WNK4 in response of rFGF23. Is total WNK immunoprecipitated equal in these lanes? AntiWNK4 (on WNK4 immunoprecipitations) should be used as a loading control.

In the text, results page 12, third line from top. "Furthermore, co-immunoprecipitation experiments..." referred to figure 7D: I see no co-precipitation experiments between two different molecules here, but rather a simple immunoprecipitation.

1st Revision - authors' response 20 August 2013

We would like to thanks the reviewers for their constructive critique.

Reviewer 1

The manuscript of Andrukhova et al. describes a novel mechanism for the regulation of renal calcium reabsorption by Klotho and FGF23. The authors propose that FGF23 acts on a basolaterally located FGF23-receptor containing Klotho as a co-factor to increase apical membrane expression of the calcium entry channel TRPV5 via ERK`/2, SGK1 and WNK4. This mechanism is at odds with the mechanism proposed by Chang et al. (Science, 2005), who indicated that Klotho can be present on the apical side and in urine, and acts by hydrolizing extracellular sugar residues on the TRPV5 channel. Overall, the manuscript is interesting and of high quality.

Main points:

1. It is not fully clear why some results are in discepancy with Chang et al. or whether both mechanisms could work in concert/parallel. The sentence on Page 7 "The fact that Klotho and

Fgf23 deficiency have almost identical effects on renal TRPV5 is difficult to explain on the basis of the model shown in Fig. 1A" is meaningless, as Fig. 1A does not include Fgf23 or its receptor. The fact that Klotho deficiency and Fgf23 deficiency have the same effects does not invalidate the model in Fig. 1A. As outlined below, I don't see the real evidence in this paper that the model by Chang et al. is necessarily wrong; the mechanisms could work in concert/parallel.

Authors: Fig. 1A of the manuscript is adapted from Cha et al., 2008, and does not include Fgf23 and its receptor. We explicitly state this now in the figure legend. Our point was that the model described by Chang et al. (2005) and Cha et al. (2008) is based on a Klotho-driven mechanism, regulating the apical membrane abundance of TRPV5. The latter model does not include FGF23. The current study identified an FGF23-driven regulation of TRPV5 apical membrane expression and channel activity. Both models are totally different. However, we agree that both mechanisms presented in Fig. 1 may exist in parallel. We explicitly state in the Discussion of the revised manuscript on page 15 now that it is still possible that Klotho has additional enzymatic functions beyond its function as coreceptor for FGF23. This could well be.

2. The only actual argument against the mechanism proposed by Chang et al. is that Andrukhova et al. fail to detect Klotho in urine (i.e. compare Fig. 2D with Fig. 1b in Chang et al.). There is no clear discussion why this difference could occur (differences in experimental protocol, mouse strain, antibody??), nor is there any positive control that the authors can detect any protein in WB on mouse urine. There are also other authors that found Klotho in urine using wb or ELISA. This point should be further discussed and positive controls of WB on urinary proteins should be included (e.g. uromodulin).

Authors: We do not have a good explanation for this discrepancy. However, differences in the antibodies used may account for the observed differences. We included more controls in the revised manuscript (Supplementary Fig. 1). While urinary uromodulin was readily detected, we were unable to detect transmembrane or ectodomain shed forms of Klotho protein by Western blotting in native, salt precipitated, or concentrated urine of our C57BL/6 mice. The two anti-Klotho antibodies used in the present study appear to be very specific, because they give no signal in tissues from Klotho deficient mice, neither in immunoblots nor in immunohistochemistry and immune-electron microscopy. We included a statement on page 7 of the revised manuscript that the differences between our results and those reported by other groups may be due to differences in the antibodies used.

3. The histological data (immunohistochemistry, immuno-electron microscopy) as shown are very much a "matter of belief". There is no quantification of crucial aspects such as colocalisation (e.g. Fig. 6A) or the degree of (sub)plasmalemmal localisation (e.g. Fig. 6C), nor is there an indication of the number of slides/animals that were analysed. Since these data are essential for the conclusions, some indication of the significance of the effects is essential.

Authors: We agree and have changed the manuscript accordingly. A quantification of TRPV5-WNK4 co-localization is now included in the revised Fig. 6A. The number of animals/slides/ROIs is now mentioned in M&M (Immunohistochemistry). For this analysis we used 4 animals per group, with 3-6 slides per animal in which 4-6 different ROIs were measured. Moreover, we included a quantification of the TRPV5 staining in the apical cellular compartment in the immuno-electron microscopic images. These data are presented in Fig. 6C. Detailed information about the sampling is included in M&M of the revised manuscript (Immuno-electron microscopic analysis).

4. The paper lacks direct assesment of TRPV5 function. Although extreted calcium levels and membrane staining could well be indications of TRPV5 function, this is not necessarily the case. For example, PACSIN3 causes an increase in membrane expression of the related channel TRPV4, yet leads to a reduced channel function. To demonstrate that the proposed mechanism leads to increased TRPV5 channel function, some more direct measurements (e.g. patch-clamp, calcium uptake in TRPV5-expressing cells) need to be included.

Authors: We fully agree that data about TRPV5 activity are very useful for this type of study. To this end, we performed intracellular calcium measurements in Fluo-4-loaded kidney slices from vehicle- and rFGF23 treated animals, using 2-photon microscopy. These data are shown in Fig. 4G of the revised manuscript. FGF23-treated mice showed an about 5-fold upregulation in fluorescence intensity in distal renal tubules, indicating that the FGF23-induced upregulation of TRPV5 membrane expression also augments calcium entry into distal tubular epithelium. Moreover, we reconstituted the SGK1-WNK4 signaling pathway in MDCK cells, and showed with intracellular calcium imaging in Fluo-4-loaded cells that increased FGF23-induced TRPV5 expression was associated with increased intracellular calcium concentrations (Fig. 8). The increased cellular calcium uptake could be blocked by the specific TRPV5 inhibitor ruthenium red in both kidney slices and transfected MDCK cells (Fig. 4G and 8). We thank the reviewer for this suggestion which has significantly strengthened the manuscript.

Reviewer 2

This reviewer supports the concept and conclusions described in this manuscript. The followings are commented to strengthen the concept and conclusions of authors.

Major points
1. The authors observed that urinal calcium excretion levels of alpha-Klotho (aKl)/VDR-double knockout-mice(KOM) and FGF23/VDR-double KOM are larger than that of VDR-single KOM. This fact possibly indicates that aKl/FGF23 signal makes a distinct effect rather than vitamin D suppression. Thus, the authors hypothesized that FGF23/aKl signaling pathway induces both TRPV5 expression and translocation to the apical lumen of the tubule. The reviewer has the following questions and comments.

1. The authors should show the general data of aKl/VDR-double KOM, FGF23/VDR-double KOM and VDR-single KOM, including Ca, Pi, PTH, VD3, calcitonin, FGF23, and bone mineral density. Based on severer impairment in Ca reabsorption, there may be different profile of mineral index between double- and single-KOM. Describe the details and discuss that point.

Authors: This is actually a very good point. We have all the data mentioned by the reviewer. As a matter of fact, $Fgf23^{-/-}/VDR^{\Delta/\Delta}$ as well as in $Kl^{-/-}/VDR^{\Delta/\Delta}$ mutants develop secondary hyperparathyroidism and show reduced bone mineral density due to chronic renal calcium wasting as well as renal PTH resistance. Although certainly very interesting, we did not include these data in the current manuscript because this would require an additional in-depth discussion of the interaction between PTH and FGF23 signaling. Overall, we think that this would distract the reader from the central aspect of this study, namely the regulation of renal calcium reabsorption by FGF23. The interaction between PTH and FGF23 signaling will be presented in a separate manuscript which is already in preparation. We hope that this is acceptable.

2. In general, urinary calcium reabsorption depends on a variety of cation channels in the tubular segments. The weakest point of the manuscript is that the authors did not address whether abundant urinary Ca excretion in double-KOM directly depends on TRPV5 translocation in the tubule cells. The authors should explain how they concluded TRPV5 as the cause of urinary Ca excretion.

Authors: It is well known that TRPV5 is the rate-limiting step in transcellular calcium transport in distal tubular epithelium. In addition, it was reported that Klotho regulates TRPV5 activity (Chang et al, 2005). Therefore, we focused on TRPV5. Moreover, we included ex vivo intracellular calcium imaging data in the revised manuscript, employing 2-photon microscopy (Fig. 4G). These data clearly show that the specific TRPV5 inhibitor ruthenium red profoundly reduces calcium uptake in

distal tubules of FGF23-treated mice, showing that the FGF23-induced stimulation of distal tubular calcium uptake is mainly caused by TRPV5 (page 9 of revised manuscript).

3. The authors proposed that FGF23/aKlotho signaling induces TRPV5 translocation through pERK1/2, pSGK1, WNK4 kinase pathway. The pathway seems reasonable. To strengthen this hypothesis, it would be better that the authors reconstitute TRPV5 translocation system using the relevant signaling molecules in cultured cells such as MDCK.

Authors: We reconstituted the SGK1-WNK4 pathway in MDCK cells as suggested. We transfected MDCK cells with mouse TRPV5, WNK4 and SGK1 constructs and treated the cells with recombinant FGF23 (rFGF23) alone or in combination with recombinant Klotho (rKL). MDCK cells are known to express ERK1/2 and FGF receptor 1, also involved in FGF23 signaling. The data are shown in Fig. 8 of the revised manuscript. Treatment with rFGF23 significantly increased the expression of complex glycosylated TRPV5 in MDCK cells transfected with TRPV5, WNK4 and SGK1. In the absence of SGK1, rFGF23 was without effect. In the absence of WNK4, we still saw a small increase in TRPV5 expression which may be due to low default expression of WNK kinases in MDCK cells. Additionally, we show by 2-photon microscopy that rFGF23 treatment increased intracellular calcium in MDCK transfected with TRPV5, WNK4 and SGK1 constructs. This effect was blocked by the specific TRPV5 inhibitor ruthenium red. We feel that these additional data provide strong evidence in support of our hypothesis that FGF23 regulates TRPV5 expression by a signaling pathway involving SGK1 and WNK4.

4. The authors observed TRPV5 intracellular location of the tubular cells mainly using immuno-histochemistry (Figure3,4) and the histological images were used for the chief evidences to demonstrate aKl/FGF23-depedent TRPV5 translocation system. However, unclear images did not necessarily address the convincing conclusion. The authors have to obtain clearer images using rational control, such as increased calbindin D and NaPi2c translocation to the plasma membrane on vitamin D and FGF23 injection, respectively. EM images of Figure 6 also could not succeed in clarifying the inter-organellar transport (as described in page 11).

Authors: To clarify the inter-organellar transport of TRPV5 and WNK4 after rFGF23 treatment we included new higher magnification immuno-electron microscopy images in Fig. 6B of the revised manuscript. In addition, we performed immuno-electron microscopic analysis of calbindin-D-28k in kidneys from vehicle- and rFGF23-treated animals. The anti-calbindin-D-28k immuno-electron microscopy data are included in Supplementary Fig. 3. Although calbindin D28k mRNA abundance was increased 8 hours after rFGF23 administration, no clear difference in the intracellular distribution pattern was detected between vehicle and rFGF23 treatment. We did not analyze NaPi2c translocation because this molecule is only expressed in proximal tubules.

Minor point
1. In Figure 4 B, serum PTH of VDR-KOM is maintained at approximately 50pg/ml. Even fed with rescue diet, it seems curious that the PTH level is completely restored. In VDR-KO state, dehydration will be quietly enhanced for abundant Ca excretion. For example, mineral contents of supplied water need to be checked, if the authors did not choose deionized water.

Authors: The serum PTH data in Fig. 4B are from wild type, not from VDRKO animals.

2. In addition, the authors are requested to explain the phenomenon that vehicle administration resulted in continuous PTH secretion.

Authors: This is actually a good point. The reason for the increase in serum PTH in vehicle-treated animals was that we performed this experiment in animals killed immediately after an overnight fast in metabolic cages. The fasting probably up-regulated serum PTH. The rise in serum PTH was suppressed by rFGF23 treatment. We repeated this experiment in rFGF23- and vehicle-treated mice kept in their normal cage environment with free access to food. The new data are shown in Fig. 4B of the revised manuscript. No significant difference in serum PTH levels between vehicle and rFGF23 treatment was found.

Reviewer 3

Previous studies based mostly on in vitro assays had proposed that klotho could have an effect on the activity of the TRPV5 channel by directly and physically regulating the glyosylation state of TRPV5 thanks to its putative glycosidase activity.
Andrukhova et al now use a series of animal models to elegantly study the cross-talks between klotho, the TRPV5 channel and regulation of Calcium homeostasis in the distal tubules of the kidney. The results provided suggest an alternative explanation for previous findings which appears more in line with the accumulating evidence of klotho acting mainly as a co-receptor for FGFR in response to FGF23 stimutaion.

The study is certainly worth of notice as it builds on a careful, systematic and accurate analysis of the system in physiological conditions which is likely to provide a credible mechanistic explanation for the cross-talk between Klotho and the TRPV5, which appears to be indirect via signaling of SGK1 and WNK4.

This reviewer has two main concerns with the data:

1. The piece of data which appears to be key and clearly highlights the need for proposing the model shown in Figure 1B as opposed to the model proposed in Figure 1A is the subcellular localization of klotho. The authors use nicely controlled studies of Kl-/- tissues to characterize two distinct antibodies directed against klotho. The western blot analysis is convincing and shows specific immunodetection. On the basis of this specificity in western blot analysis the data showing lack of klotho in the urines (Supplementary Figure 3D) are very important. Could the authors attempt at performing protein enrichment in the urine such as salt precipitation or volume decrease to make sure that minimal amounts of klotho are not detectable in the urines?

Authors: We included additional anti-Klotho Western blotting analyses of urine samples which were protein-enriched by salt precipitation and ultrafiltration, as suggested. These additional data are shown in Supplementary Fig. 1 of the revised manuscript. We also included additional control immunoblots using anti-uromodulin antibody. However, we were unable to detect any positive signal in urine samples with both anti-Klotho antibodies used in our study.

The immunolocalization studies shown in Figure 3B do not appear very specific, as the staining appears to be rather diffused. Having in hands klotho-/- tissues, again, could the authors attempt at performing immuno-EM localization of klotho as a more precise evidence of its basal localization?

Authors: We performed immuno-electron microscopic analysis of Klotho localization in renal distal tubules, using two different anti-Klotho antibodies, as suggested. As shown in Fig. 3B of the revised manuscript both anti-Klotho antibodies showed Klotho expression in the basolateral membrane of distal tubular cells. Staining was absent in the apical membrane. Kidney slices from Klotho$^{-/-}$ animals were used as a negative control.

2. Second, based on their studies the authors propose that klotho regulates the TRPV5 distribution and function indirectly, acting as a co-receptor for the FGFreceptor (on the basolateral side of

© EMBO

cells, see above). The data showing similar pohenotypes in the Fgf23-/- and kl-/- animals (both on VDRdelta/delta background) indeed strongly suggest that the specific function of klotho on TRPV5 and calciuria are mediated via its classical function of co-receptor for FGF23. The obvious subsequent experiment that would definitively demonstrate this would be the generation of double mutant animals (triple in this case: Kl-/-;FGF23-/-;VDRdelta/delta) and the analysis of the phenotype which should be identical to the individual mutants (Kl-/- and FGF23-/-, respectively) if indeed their interaction is the the only mechanism acting here.

Authors: This is actually a very good and insightful suggestion. We tried hard to do this in the last months. However, the breeding yielded only one $Fgf23^{-/-}$/$Klotho^{-/-}$/$VDR^{\Delta/\Delta}$ triple mutant animal so far. In line with our hypothesis, this animal displayed no difference in gross phenotype and unchanged blood ionized calcium concentration as compared to $VDR^{\Delta/\Delta}$ single mutants, as well as $FGF23^{-/-}$/ $VDR^{\Delta/\Delta}$ and $Klotho^{-/-}$/ $VDR^{\Delta/\Delta}$ double mutant littermates. Other data are currently not available. We did not include these data because the number of animals per group is clearly insufficient (n = 1 – 3 per group). However, we are mentioning these preliminary data and our attempt to generate triple KO mice in the Discussion of the revised manuscript on page 16. The breeding is difficult to do, because for an effective breeding strategy, one has to do the breeding on VDR mutant background. Even on rescue diet, lactation is a challenge for VDR deficient dams. Therefore, all the pups have to be given to foster mothers. It is very hard to predict how long it will take to complete the groups. Although the data would certainly be very interesting and would further strengthen the manuscript, we suggest not to postpone publication of the current manuscript any longer.

Minor:

1.Both in figure 5D and 7D, the loading control is not povided. Immunoprecipitation of WNK4 followed by Immunoblotting with anti-P-ser shows increased phosphorylation of WNK4 in response of rFGF23. Is total WNK immunoprecipitated equal in these lanes? AntiWNK4 (on WNK4 immunoprecipitations) should be used as a loading control.

Authors: This is a good point. We have changed Figs. 5E and Fig. 7D of the revised manuscript accordingly. There were no differences in WNK4 immunoprecipitation.

2. In the text, results page 12, third line from top. "Furthermore, co-immunoprecipitation experiments..." referred to figure 7D: I see no co-precipitation experiments between two different molecules here, but rather a simple immunoprecipitation.

Authors: We have changed the text accordingly in the revised manuscript. Thank you.

Editorial Decision 16 September 2013

Thank you for submitting a revised version of your manuscript to The EMBO Journal. It has now been seen by two of the original referees whose comments are shown below.

As you will see, while referee #2 finds that the manuscript has been significantly improved and in principle supports publication, referee #1 is not convinced by the experimental data added to address TRPV5 function. In particular, the referee points out that the use of Ruthenium Red cannot be used as a measure for specific TRPV5 inhibition and that it may in addition interfere with detection of Fluo-4 signals. Furthermore, ref#1 asks for dynamic, rather than steady state, assessment of Calcium signaling. While these are serious concerns that will have to be addressed before we can take any further steps towards publication of your manuscript, referee #1 does remains supportive of your study, pending the inclusion of more decisive control experiments.

Given the overall recommendations from the referees, I would like to invite you to submit a revised version of the manuscript, addressing the specific criticisms raised by ref #1. In order to facilitate the process, I would also already at this stage ask you to provide me with an outline of the experiments that you would be able to include in a revised manuscript. I will then consult with ref #1 to clarify whether the outlined experiments would suffice to address the remaining concerns and keep you informed before you initiate the required additional work.

Please feel free to contact me with any questions concerning this matter and thank you for the opportunity to consider your study for The EMBO Journal. I look forward to your revision!

REFEREE REPORTS:

Referee #1:

The authors have made important improvements to the manuscript. However, in my opinion the newly included data to assess TRPV5 function are not adequate and potentially flawed.

First, in contrast to what the authors write in the manuscript, Ruthenium Red is NOT a specific TRPV5 inhibitor. It blocks every TRPV channel and has many more targets. In no way can it be used to separate TRPV5 function.

Second, Ruthenium Red is red, and may absorb significant parts of the Fluo-4 spectrum, which may (at least partly) explain reduced Fluo-4 signals in the treated cells/tissue. No controls are included to assess/exclude this.

Third, since only steady-state fluorescence signals are measured, they do not represent a measure of channel activity but at most a measure of steady-state cytosolic calcium, which could just be dependent on pumps/buffers. Changes could also be merely due to differences in the concentration of loaded Fluo-4. One could look at changes in fluorescence in response to changes in extracellular calcium, to have an indication whether channel activity is changed.

In my opinion, these new data do not strengthen the paper and are potentially flawed, and the question whether TRPV5 activity is altered remains open. Nevertheless,I am convinced that the authors can provide better data, e.g. with better controlled calcium imaging data in MDCK cells, to support their mechanism.

Referee #2:

Re-submitted manuscript is well revised. Particularly, authors succeeded to re-constitute FGF23/aKlotho signaling system that induces TRPV5 translocation through pERK1/2, pSGK1, WNK4 kinase pathway and thus authors' conclusions were strengthened. Therefore, this reviewer supports the concept and conclusions described in this manuscript. This paper should be published in this Journal.

Additional Author Correspondence: 20 September 2013

Please find attached our response to reviewer 1, the revised manuscript, revised Fig. 4, 4 videos, and the brief legends for the videos.

Response to reviewers

We would like to thank the reviewer for the constructive critique.

Reviewer 1

The authors have made important improvements to the manuscript. However, in my opinion the newly included data to assess TRPV5 function are not adequate and potentially flawed.

First, in contrast to what the authors write in the manuscript, Ruthenium Red is NOT a specific TRPV5 inhibitor. It blocks every TRPV channel and has many more targets. In no way can it be used to separate TRPV5 function.

Authors: The point that ruthenium red is not a specific TRPV5 inhibitor is certainly valid. Thank you. It is clear that ruthenium red inhibits all TRPV channels and also other ion channels. The reason why we and others (e.g. van Abel et al., Kidney Int 68:1708-21, 2005) called ruthenium red a specific TRPV5 inhibitor is that TRPV5 is about 100-fold more sensitive to ruthenium red than TRPV6 (Hoenderup et al., J Physiol 537:747, 2001). For distal tubular calcium absorption, only TRPV5 and 6 are thought to be relevant. Therefore, with regard to distal tubular calcium transport and relative to TRPV6, ruthenium red has been used as a ‚TRPV5 specific‘ inhibitor at concentrations up to about 10 µM. However, this statement is certainly not true in general terms. We have amended the manuscript to be clear about this, and have deleted the term 'TRPV5 specific'. Apart from TRPV5 and TRPV6, the only other TRPV channel expressed in distal renal tubules may be TRPV4. TRPV4 and 5 have similar sensitivity to ruthenium red. Earlier data suggested that TRPV4 is also expressed in distal tubular epithelium (reviewed in Woudenberg-Venken et al., Nat Rev Nephrol 5:441, 2009). However, more recent studies suggested that the main site of TRPV4 expression is the collecting duct (Berrout et al., JBC 287:8782, 2012). TRPV4 functions as osmosensor and mechanosensor for fluid flow, but has no known role in transcellular calcium transport (Pochynyuk et al., Pflugers Arch 465:177, 2013). To further demonstrate the regulation of TRPV5 activity by FGF23, we have included additional data in the revised manuscript, showing that rFGF23 increases distal tubular intracellular calcium concentrations in kidney slices *in vitro*, and that this effect can be reversed by ruthenium red at concentrations as low as 1 µM (Fig. 4H). Ten and 50 µM of ruthenium red did not further decrease the fluorescence signal, showing that TRPV6 does not play a major role in the FGF23-induced increase in calcium uptake in distal tubular cells (Fig. 4H). At 1 µM, ruthenium red does not antagonize TRPV6 function. Full inhibition of TRPV6 requires ruthenium red concentrations beyond 30 - 50 µM. We feel that these additional data lend further support to our conclusion that FGF23 activates mainly TRPV5, not TRPV6, in distal tubules.

Second, Ruthenium Red is red, and may absorb significant parts of the Fluo-4 spectrum, which may (at least partly) explain reduced Fluo-4 signals in the treated cells/tissue. No controls are included to assess/exclude this.

Authors: This is a good point. In principle, we agree that ruthenium red could quench the green fluorescence of Fluo-4 because of overlap of the absorption and emission spectra, respectively. However, in practice this potential problem does not interfere with the measurements. As shown below, addition of 10 µM ruthenium red does not decrease the Fluo-4 signal within the first minutes in kidney slices from rFGF23-treated mice. As an additional control, we have included Supplementary videos in the revised version, showing that directly after addition of ruthenium red the fluorescence signal is unchanged. The decrease in distal tubular intracellular fluorescence develops over 15 - 30 min after addition of ruthenium red (see Supplementary videos and Fig. 4H). Any quenching effect would appear immediately.

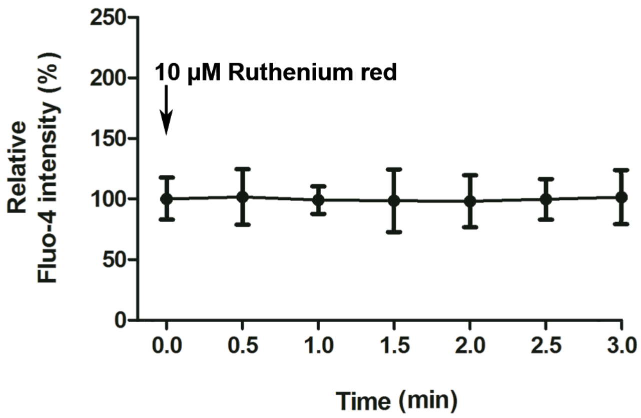

Fig. 1. Fluo-4 fluorescence intensity measured in distal tubules of Fluo-4-loaded kidney slices from rFGF23-treated mice, before (time 0) and after addition of 10 µM ruthenium red. Data are mean ± SEM.

Third, since only steady-state fluorescence signals are measured, they do not represent a measure of channel activity but at most a measure of steady-state cytosolic calcium, which could just be dependent on pumps/buffers. Changes could also be merely due to differences in the concentration of loaded Fluo-4. One could look at changes in fluorescence in response to changes in extracellular calcium, to have an indication whether channel activity is changed.

Authors: As a matter of fact, all fluorescence measurements performed using 2-photon microscopy in our study are dynamic measurements. Pictures were taken every 30 sec for all measurements. Therefore, although we took certain time points in Figs. 4G and 8B to illustrate our points, the measurements do not represent steady state intracellular calcium concentrations. All kidney slices and MCDK cell preparations were loaded with Fluo-4 in the exactly the same fashion, and washed twice for 15 min. Variability in Fluo-4 loading is not an issue as can be judged by the low standard deviations in vehicle- and rFGF23-treated mice and cells shown in Figs. 4G&H and 8B, respectively. To make the data more convincing, we have included Supplementary videos, showing i) the increase in distal tubular Fluo-4 fluorescence over two hours in exactly the same site after addition of rFGF23 in Fluo-4-loaded kidney slices from wild-type mice (Supplementary video 3), and ii) the decrease in fluorescence intensity at the same site over time after addition of ruthenium red (Supplementary videos 1 and 4). These changes in fluorescence intensity over time cannot be explained by differences in Fluo-4 loading.

In my opinion, these new data do not strengthen the paper and are potentially flawed, and the question whether TRPV5 activity is altered remains open. Nevertheless, I am convinced that the authors can provide better data, e.g. with better controlled calcium imaging data in MDCK cells, to support their mechanism.

Authors: Thank you for the suggestion. In TRPV5-transfected MDCK cells specificity of the channel inhibitor is not an issue, because the cells only express TRPV5 in this setting. To show that FGF23 activates the TRPV5 channel, we additionally performed experiments in which we treated TRPV5/SGK1/WNK4-transfected MDCK cells with rFGF23 at time 0, and followed Fluo-4 fluorescence over 1 hour. As shown in Fig. 2, rFGF23 induced a clear increase in Fluo-4 fluorescence in the transfected cells over the 1-hour time period. Ruthenium red antagonized this effect within 30 min after addition. Unless requested, we do not plan to include the latter data in the manuscript, because the abovementioned additional experiments in kidney slices (Fig. 4H) basically show the same thing, namely that rFGF23 increases the cellular uptake of calcium in distal tubules

in vitro. Collectively, we feel that these additional data provide strong evidence in favor of the notion that FGF23 upregulates TRPV5 activity.

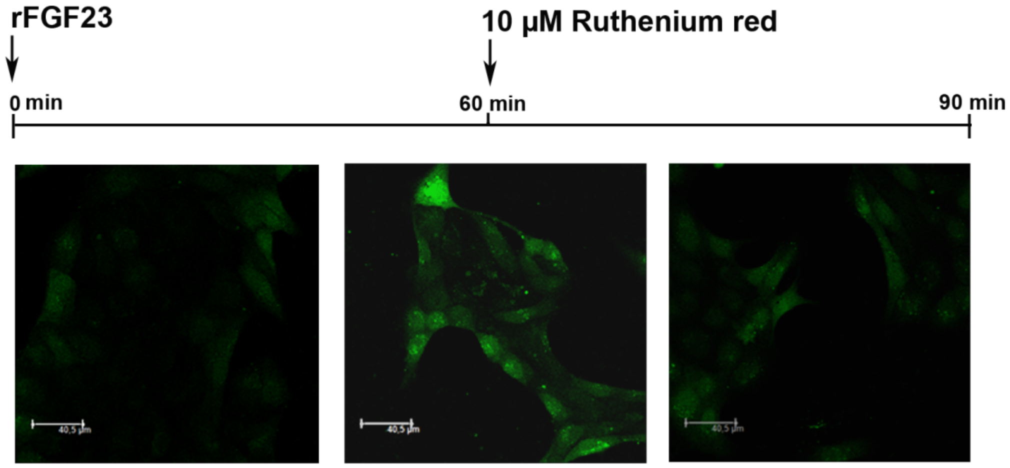

Fig. 2. MDCK cells transiently transfected with murine TRPV5, SGK1 and WNK4 constructs were treated with rFGF23 at time 0. Before rFGF23 treatment, the cells were loaded with Fluo-4 and washed two times. The fluorescent images show representative cells at time 0 (before rFGF23 treatment), 1 hour after addition of rFGF23 to the culture medium, and 30 min after addition of 10 µM ruthenium red.

Additional Editorial Correspondence 15 October 2013

We have now heard back from the referee concerning your proposal for a revised manuscript (full comment included below); however, I am sorry to say that the referee at this stage remains unconvinced that the additional data that you propose to include would suffice to specifically measure TRPV5 activity.

In essence, the referee points out that better data quality and more controls would be required and lists the following examples:
- The MDCK data included in the response to the review (fig 2) appear to derive from different experiments rather than a time-course of the same sample
- The included movies are hard to interpret and need to be more carefully analysed.
- A vehicle control should be included for the FGF23 application in fig 4H
- An experiment involving the removal of extracellular Ca2+ is required to convincingly establish the effect on a calcium influx pathway.

Furthermore, the referee remains skeptical about the effects of RR addition, given the discrepancy between the strongly reduced fluorescence at micromolar RR in fig 4H with the partial inhibition at much higher doses in fig 4G.

Based on these consistent criticisms from the referee, I have to ask you to address the points raised above through additional experimentation. I do appreciate that the physiological expression pattern of TRPV5 goes a long way in establishing a functional specificity for RR in blocking Calcium signaling, but I also have to agree with the reviewer that a possible contribution from other Ca2+ transporters should be formally eliminated in order to conclude that the effects of Fgf23 application are exclusively dependent on TRPV5 activation.

I would like to ensure you that the referee remains supportive of your manuscript and recommends that it should be published in The EMBO Journal, provided that these last remaining issues can be

resolved.

REFEREE REPORT:

Referee 1:
I am sorry to say that I am not very convinced by what the authors show in their response.

My main point was that TRPV5 activity was not well analyzed/quantified. What I still miss are at least some decent and statistically analyzed data sets with calcium imaging. The authors now provide some movies and still images, but these are not really analyzed and hard to interpret. For instance, the MDCK data (Fig. 2 in the letter) seem to illustrate different time points in an experiment, but upon scrutinizing the three pictures are obviously from different cells/preparations. Likewise, the pictures in Figure 4G are from different preparations, so it is difficult to interpret/exclude factors other than calcium (Fluo-4 loading or cleavage of AM, for instance).

For each experiment, I would expect to see a time course of fluorescence (e.g. like the one provided in Fig. 4H) along with the proper controls (e.g. for Fig. 4H, it is imperative to include a vehicle control for the rFGF23 application). The authors should also illustrate the effect of removing extracellular calcium, which is a minimal requirement to demonstrate that they are looking at a calcium influx pathway.

Also the RR-effects are still unclear and puzzling. For instance, why does RR (at 1 micromolar) abruptly reduce fluorescence to very low levels (way below control) in Fig. 4H, whereas a ten times higher dose causes only a partial inhibition in Fig. 4G.

The whole discussion about TRPV6 is also not very convincing. Clearly, in Fig. 4H, 1 micromolar virtually abolished the signal, so it comes as no surprise that higher concentrations have no effect, but this does not allow to exclude a contribution of TRPV6, which by the way can form heteromultimers with TRPV5 with intermediate RR sensitivities.

In summary, the authors should provide better quality of calcium imaging, with well-analyzed time courses along with proper controls to convince the readers that they are actually measuring TRPV5 activity.

Revision received 30 October 2013

Response to reviewers,

We would like to thank the reviewer for the constructive critique.

Reviewer 1

The authors have made important improvements to the manuscript. However, in my opinion the newly included data to assess TRPV5 function are not adequate and potentially flawed.

First, in contrast to what the authors write in the manuscript, Ruthenium Red is NOT a specific TRPV5 inhibitor. It blocks every TRPV channel and has many more targets. In no way can it be used to separate TRPV5 function.

Authors: The point that ruthenium red is not a specific TRPV5 inhibitor is certainly valid. Thank you. It is clear that ruthenium red inhibits all TRPV channels and also other ion channels. The

reason why we and others (e.g. van Abel et al., Kidney Int 68:1708-21, 2005) called ruthenium red a specific TRPV5 inhibitor is that TRPV5 is about 100-fold more sensitive to ruthenium red than TRPV6 (Hoenderup et al., J Physiol 537:747, 2001). For distal tubular calcium absorption, only TRPV5 and 6 are thought to be relevant. Therefore, with regard to distal tubular calcium transport and relative to TRPV6, ruthenium red has been used as a ‚TRPV5 specific‘ inhibitor at concentrations up to about 10 µM. However, this statement is certainly not true in general terms. We have amended the manuscript to be clear about this, and have deleted the term ‘TRPV5 specific’. Apart from TRPV5 and TRPV6, the only other TRPV channel expressed in distal renal tubules may be TRPV4. TRPV4 and 5 have similar sensitivity to ruthenium red. Earlier data suggested that TRPV4 is also expressed in distal tubular epithelium (reviewed in Woudenberg-Venken et al., Nat Rev Nephrol 5:441, 2009). However, more recent studies suggested that the main site of TRPV4 expression is the collecting duct (Berrout et al., JBC 287:8782, 2012). TRPV4 functions as osmosensor and mechanosensor for fluid flow, but has no known role in transcellular calcium transport (Pochynyuk et al., Pflugers Arch 465:177, 2013). To further demonstrate the regulation of TRPV5 activity by FGF23, we have included additional data in the revised manuscript, showing that rFGF23 increases distal tubular intracellular calcium concentrations in kidney slices *in vitro*, and that this effect can be reversed by ruthenium red at concentrations as low as 1 µM (Fig. 4H). Ten and 50 µM of ruthenium red did not further decrease the fluorescence signal, showing that TRPV6 does not play a major role in the FGF23-induced increase in calcium uptake in distal tubular cells (Fig. 4H). At 1 µM, ruthenium red does not antagonize TRPV6 function. Full inhibition of TRPV6 requires ruthenium red concentrations beyond 30 - 50 µM. We feel that these additional data lend further support to our conclusion that FGF23 activates mainly TRPV5, not TRPV6, in distal tubules.

Second, Ruthenium Red is red, and may absorb significant parts of the Fluo-4 spectrum, which may (at least partly) explain reduced Fluo-4 signals in the treated cells/tissue. No controls are included to assess/exclude this.

Authors: This is a good point. In principle, we agree that ruthenium red could quench the green fluorescence of Fluo-4 because of overlap of the absorption and emission spectra, respectively. However, in practice this potential problem does not interfere with the measurements. As shown below, addition of 10 µM ruthenium red does not decrease the Fluo-4 signal within the first minutes in kidney slices from rFGF23-treated mice. As an additional control, we have included Supplementary videos in the revised version, showing that directly after addition of ruthenium red the fluorescence signal is unchanged. The decrease in distal tubular intracellular fluorescence develops over 15 - 30 min after addition of ruthenium red (see Supplementary videos and Fig. 4H). Any quenching effect would appear immediately.

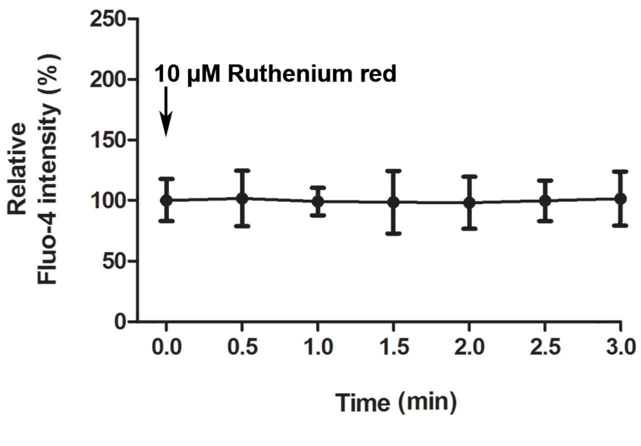

Fig. 1. Fluo-4 fluorescence intensity measured in distal tubules of Fluo-4-loaded kidney slices from rFGF23-treated mice, before (time 0) and after addition of 10 µM ruthenium red. Data are mean ± SEM.

Third, since only steady-state fluorescence signals are measured, they do not represent a measure of channel activity but at most a measure of steady-state cytosolic calcium, which could just be dependent on pumps/buffers. Changes could also be merely due to differences in the concentration of loaded Fluo-4. One could look at changes in fluorescence in response to changes in extracellular calcium, to have an indication whether channel activity is changed.

Authors: As a matter of fact, all fluorescence measurements performed using 2-photon microscopy in our study are dynamic measurements. Pictures were taken every 30 sec for all measurements. Therefore, although we took certain time points in Figs. 4G and 8B to illustrate our points, the measurements do not represent steady state intracellular calcium concentrations. All kidney slices and MCDK cell preparations were loaded with Fluo-4 in the exactly the same fashion, and washed twice for 15 min. Variability in Fluo-4 loading is not an issue as can be judged by the low standard deviations in vehicle- and rFGF23-treated mice and cells shown in Figs. 4G&H and 8B, respectively. To make the data more convincing, we have included Supplementary videos, showing i) the increase in distal tubular Fluo-4 fluorescence over two hours in exactly the same site after addition of rFGF23 in Fluo-4-loaded kidney slices from wild-type mice (Supplementary video 3), and ii) the decrease in fluorescence intensity at the same site over time after addition of ruthenium red (Supplementary videos 1 and 4). These changes in fluorescence intensity over time cannot be explained by differences in Fluo-4 loading.

In my opinion, these new data do not strengthen the paper and are potentially flawed, and the question whether TRPV5 activity is altered remains open. Nevertheless, I am convinced that the authors can provide better data, e.g. with better controlled calcium imaging data in MDCK cells, to support their mechanism.

Authors: Thank you for the suggestion. In TRPV5-transfected MDCK cells specificity of the channel inhibitor is not an issue, because the cells only express TRPV5 in this setting. To show that FGF23 activates the TRPV5 channel, we additionally performed experiments in which we treated TRPV5/SGK1/WNK4-transfected MDCK cells with rFGF23 at time 0, and followed Fluo-4 fluorescence over 1 hour. As shown in Fig. 2, rFGF23 induced a clear increase in Fluo-4 fluorescence in the transfected cells over the 1-hour time period. Ruthenium red antagonized this effect within 30 min after addition. Unless requested, we do not plan to include the latter data in the manuscript, because the abovementioned additional experiments in kidney slices (Fig. 4H) basically show the same thing, namely that rFGF23 increases the cellular uptake of calcium in distal tubules *in vitro*. Collectively, we feel that these additional data provide strong evidence in favor of the notion that FGF23 upregulates TRPV5 activity.

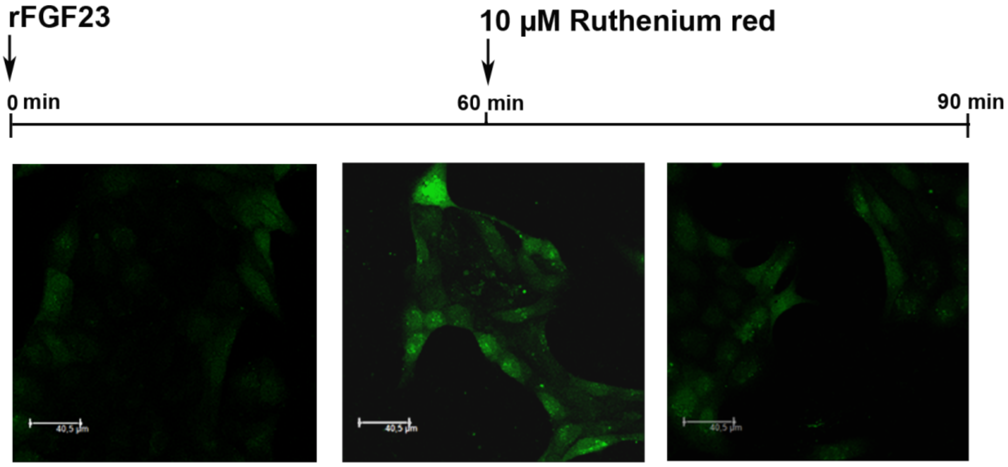

Fig. 2. MDCK cells transiently transfected with murine TRPV5, SGK1 and WNK4 constructs were treated with rFGF23 at time 0. Before rFGF23 treatment, the cells were loaded with Fluo-4 and washed two times. The fluorescent images show representative cells at time 0 (before rFGF23 treatment), 1 hour after addition of rFGF23 to the culture medium, and 30 min after addition of 10 µM ruthenium red.

Second response

Reviewer 1

My main point was that TRPV5 activity was not well analyzed/quantified. What I still miss are at least some decent and statistically analyzed data sets with calcium imaging. The authors now provide some movies and still images, but these are not really analyzed and hard to interpret. For instance, the MDCK data (Fig. 2 in the letter) seem to illustrate different time points in an experiment, but upon scrutinizing the three pictures are obviously from different cells/preparations. Likewise, the pictures in Figure 4G are from different preparations, so it is difficult to interpret/exclude factors other than calcium (Fluo-4 loading or cleavage of AM, for instance).

Authors: We don't understand the first part of the comment. The movies exemplarily show that intracellular Fluo-4 fluorescence in distal tubules is increased after administration of FGF23, and decreases after addition of RR at specific sites. State-of-the-art quantification and statistical analysis of the intracellular calcium imaging data is already provided in Figs. 4G, 4H, and 8B. It is not true that the MDCK data in Fig. 2 in our previous response to reviewers are from different experiments. All pictures are from the same time course experiment. It is just not the identical cells, but they are all from the same well. The pictures in Fig. 4G (Veh vs. FGF23) must be from different animals. This is inherent in the experimental design. We added the movie to show that fluorescence decreases after addition of RR over time at exactly the same site, in addition to the quantification shown in Fig. 4G.

For each experiment, I would expect to see a time course of fluorescence (e.g. like the one provided in Fig. 4H) along with the proper controls (e.g. for Fig. 4H, it is imperative to include a vehicle control for the rFGF23 application). The authors should also illustrate the effect of removing extracellular calcium, which is a minimal requirement to demonstrate that they are looking at a calcium influx pathway.

© EMBO

Authors: We included a vehicle control in Fig. 4H in the revised manuscript. In addition, we performed a time course experiment in MDCK cells transfected with TRPV5, SGK1 and WNK4 in which we added EGTA to the culture medium to chelate calcium. In the presence of EGTA, FGF23 did not increase intracellular Fluo-4 fluorescence, unequivocally indicating that FGF23 stimulates calcium influx (Fig. 8C).

Also the RR-effects are still unclear and puzzling. For instance, why does RR (at 1 micromolar) abruptly reduce fluorescence to very low levels (way below control) in Fig. 4H, whereas a ten times higher dose causes only a partial inhibition in Fig. 4G.

Authors: The discrepancy between Figs. 4G and H can likely be explained by the following: kidney slices in Fig. 4G are from animals treated with FGF23 8 hours before necropsy. This is an ex vivo experimental setting. Therefore, there is already pronounced upregulation of TRPV5 membrane abundance in FGF23-treated mice, because the distal tubules in these mice were pre-exposed to FGF23 for 8 hours. In addition, as shown in Fig. S3E, there is already beginning up-regulation of intracellular calcium binding proteins after 8 hours which may result in higher residual Fluo-4 fluorescence after blocking TRP channels with RR. In contrast, in Fig. 4H we treated kidney slices from wild-type mice with FGF23 in vitro for 2 hours. Transport of TRPV5 protein to the plasma membrane takes time. Therefore, this experiments is looking at very early effects of FGF23 on distal tubules. Therefore, it is easier to block the latter effect with lower concentrations of RR, compared with the ex vivo situation. The fact that the FGF23-induced increase in intracellular calcium can be completely reversed by micromolar concentrations of RR in Fig. 4H tells that this effect is mainly due to TRPV5, not TRPV6. We included a brief explanation of the differences between the experiments shown in Fig. 4G and H in the revised manuscript on page 10. The reason why RR reduced intracellular fluorescence in FGF23- and vehicle-treated kidney slices to levels below baseline (time 0) probably is that TRPV5-mediated calcium influx in distal tubular cells is an important calcium entry pathway also under normal conditions.

The whole discussion about TRPV6 is also not very convincing. Clearly, in Fig. 4H, 1 micromolar virtually abolished the signal, so it comes as no surprise that higher concentrations have no effect, but this does not allow to exclude a contribution of TRPV6, which by the way can form heteromultimers with TRPV5 with intermediate RR sensitivities.

Authors: When we treated kidney slices with FGF23 in vitro (Fig. 4H), the effect could be totally reversed by RR at 1 micromolar concentration. This tells that this effect is mainly mediated through TRPV5. Calcium uptake in distal tubules is mediated through TRPV5 and TRPV6. The IC50 of TRPV5 for RR is 0.1, that of TRPV6 10 micromolar. Therefore, at 1 uM RR, TRPV6 is not inhibited (maybe a few percent). This experiment therefore indicates that the main effect of FGF23 on distal tubular calcium uptake in vitro is mediated through TRPV5. It is true that TRPV5 and 6 can form heteromultimers with intermediate RR sensitivities. Therefore, we cannot completely exclude a small contribution of TRPV6. In vivo, the situation may be more complicated. RR at 10 μM largely, but not completely, reversed the increased intracellular fluorescence signal in kidney slices from FGF23-treated mice (Fig. 4G). Therefore, there may be a contribution of TRPV6 to the effects of FGF23 on distal tubular calcium reabsorption. We officially state now in the revised manuscript that we cannot exclude a contribution of TRPV6 to the effects of FGF23 on renal tubular calcium reabsorption (page 10).

In summary, the authors should provide better quality of calcium imaging, with well-analyzed time courses along with proper controls to convince the readers that they are actually measuring TRPV5 activity.

Authors: The quality of our calcium imaging is state-of-the-art. In addition, our experiments in transfected MDCK cells clearly show that FGF23 increases TRPV5 expression and calcium uptake via activation of SGK1 and WNK4. This is in the absence of TRPV6, and the effect can be blocked by RR. Therefore, together with our in vivo data showing that FGF23 regulates TRPV5 protein expression, renal calcium excretion, and distal tubular calcium uptake, we feel that the current study provides conclusive evidence that FGF23 regulates TRPV5 expression and function.

Accepted 18 November 2013

I am pleased to inform you that your manuscript has now been officially accepted for publication in The EMBO Journal, pending a few minor editorial points as listed below:

- Please make sure that number of replicas used for calculating statistics is indicated in figure legends (currently missing for figs 5, 7 and 8), also for supplementary figures.

- Please include scale bars in images in figs 3A, 4D and 6A and state scale bar size in corresponding figure legend.

As of Jan 1st 2014 every paper published in The EMBO Journal will include a 'Synopsis' to further enhance its discoverability. This synopsis will include a short standfirst - written by the handling editor - as well as 2-5 one-sentence bullet points that summarise the paper and are provided by the authors. These bullet points should be complementary to the abstract - i.e. not repeat the same text ->I would ask you to provide your suggestions for bullet points for this manuscript.

The amended figure legends, figures and bullet points can be sent to me by email and we will then upload everything in house.
