# Supplementary figures and images for: FGF23 promotes renal calcium reabsorption through the TRPV5 channel
Source: EMBO J. 2014 Jan 17;33(3):229–46. doi: 10.1002/embj.201284188 (PMC3983685; doi:10.1002/embj.201284188)

Figure 2.

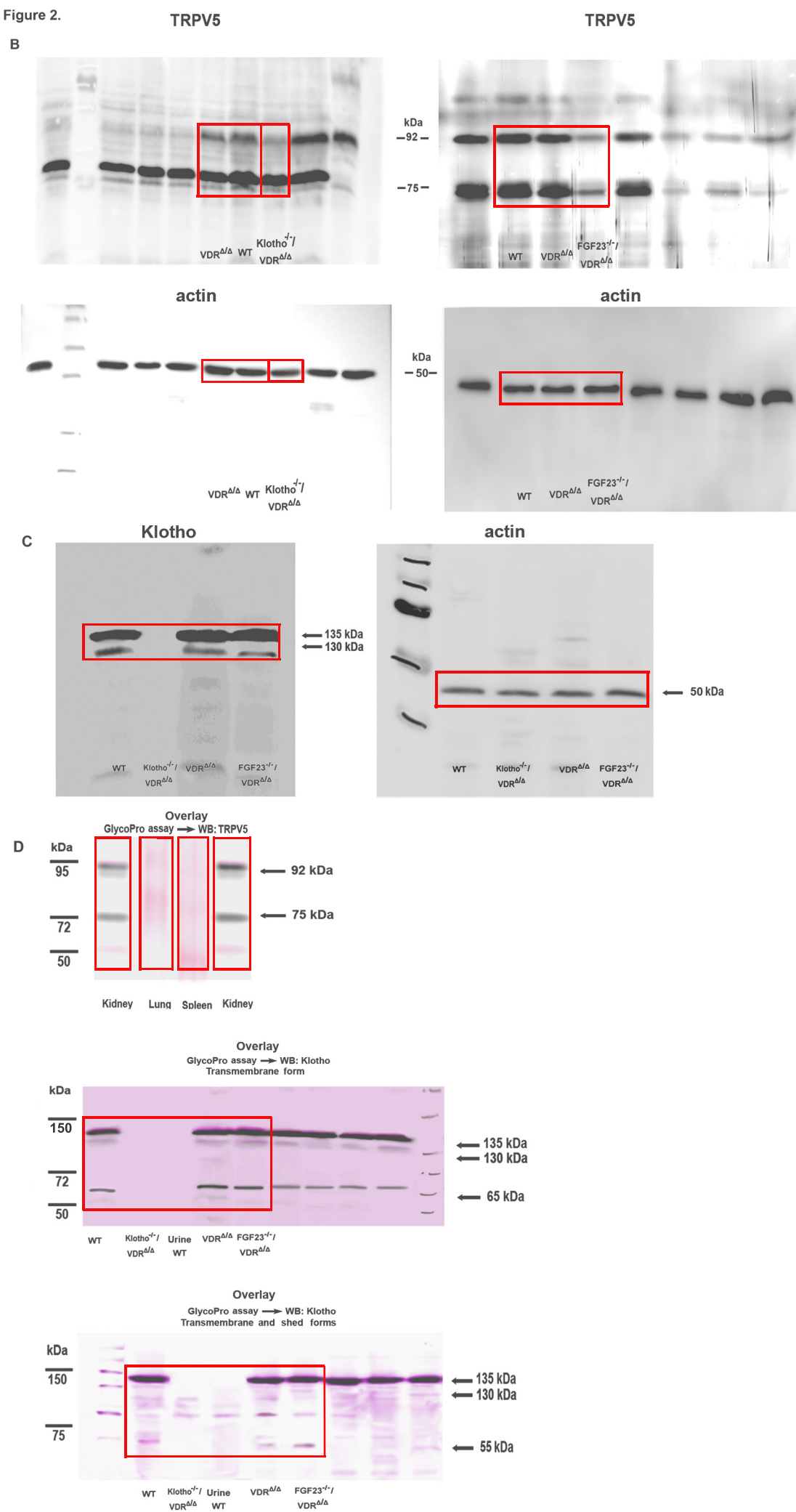

Supplement: Supplementary file 13 [file embj0033-0229-sd13.pdf]

**Figure 4.**

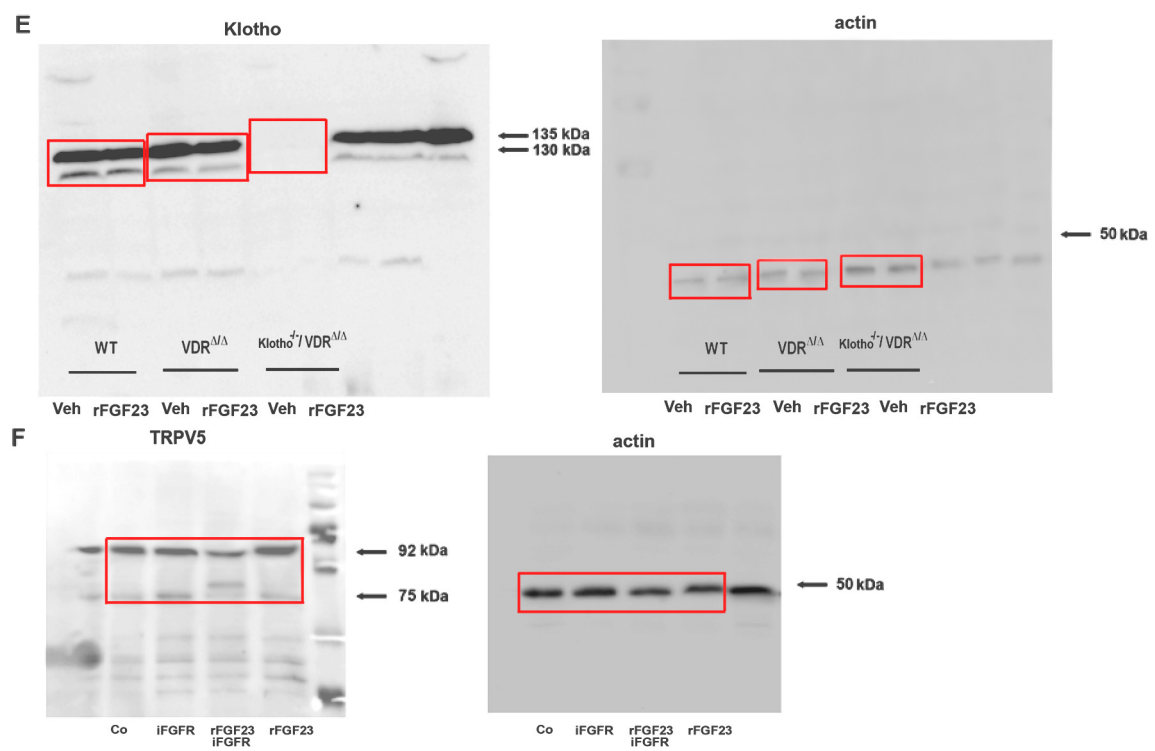

Supplement: Supplementary file 14 [file embj0033-0229-sd14.pdf]

Figure 5

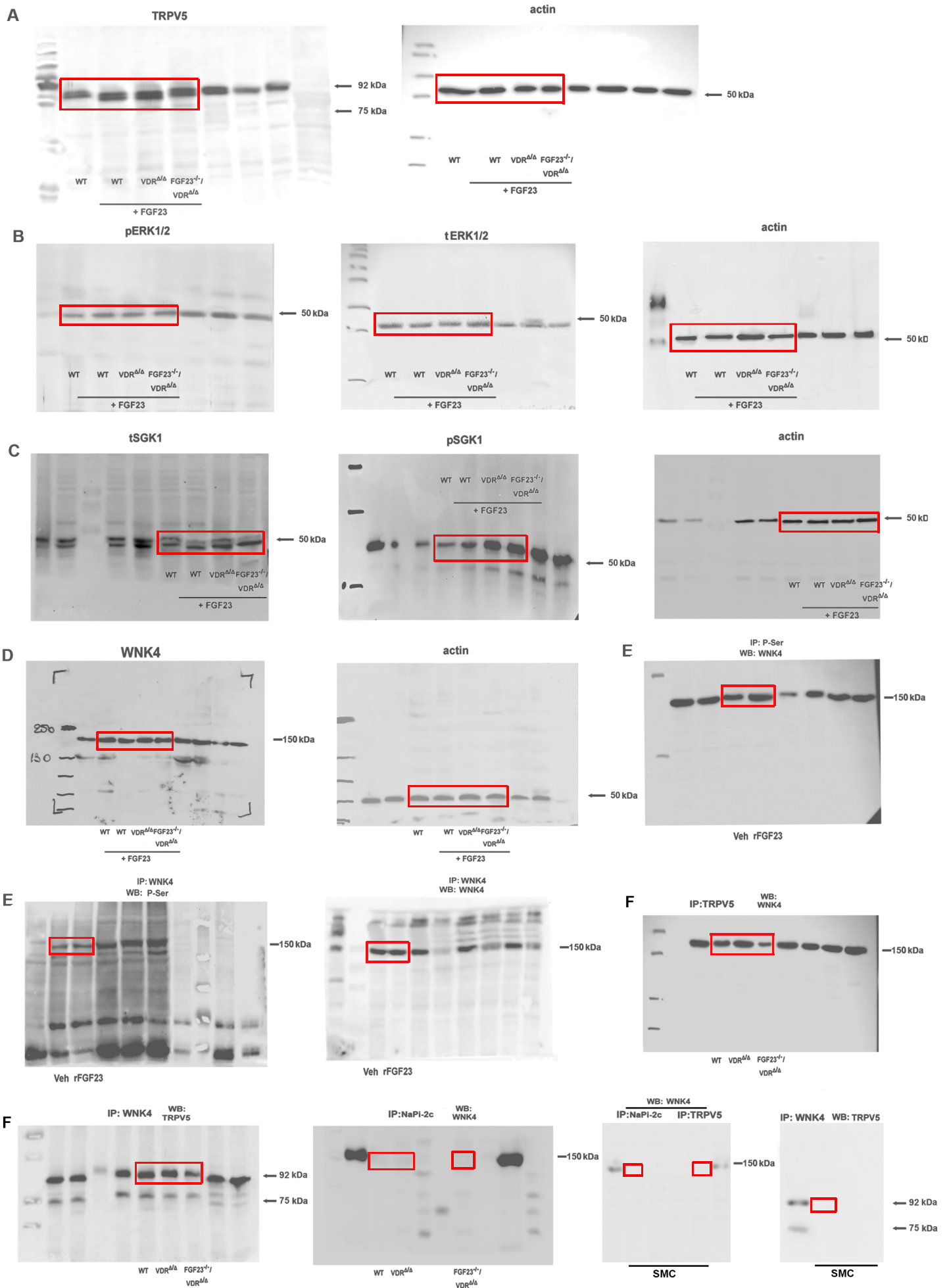

Supplement: Supplementary file 15 [file embj0033-0229-sd15.pdf]

Figure 7.

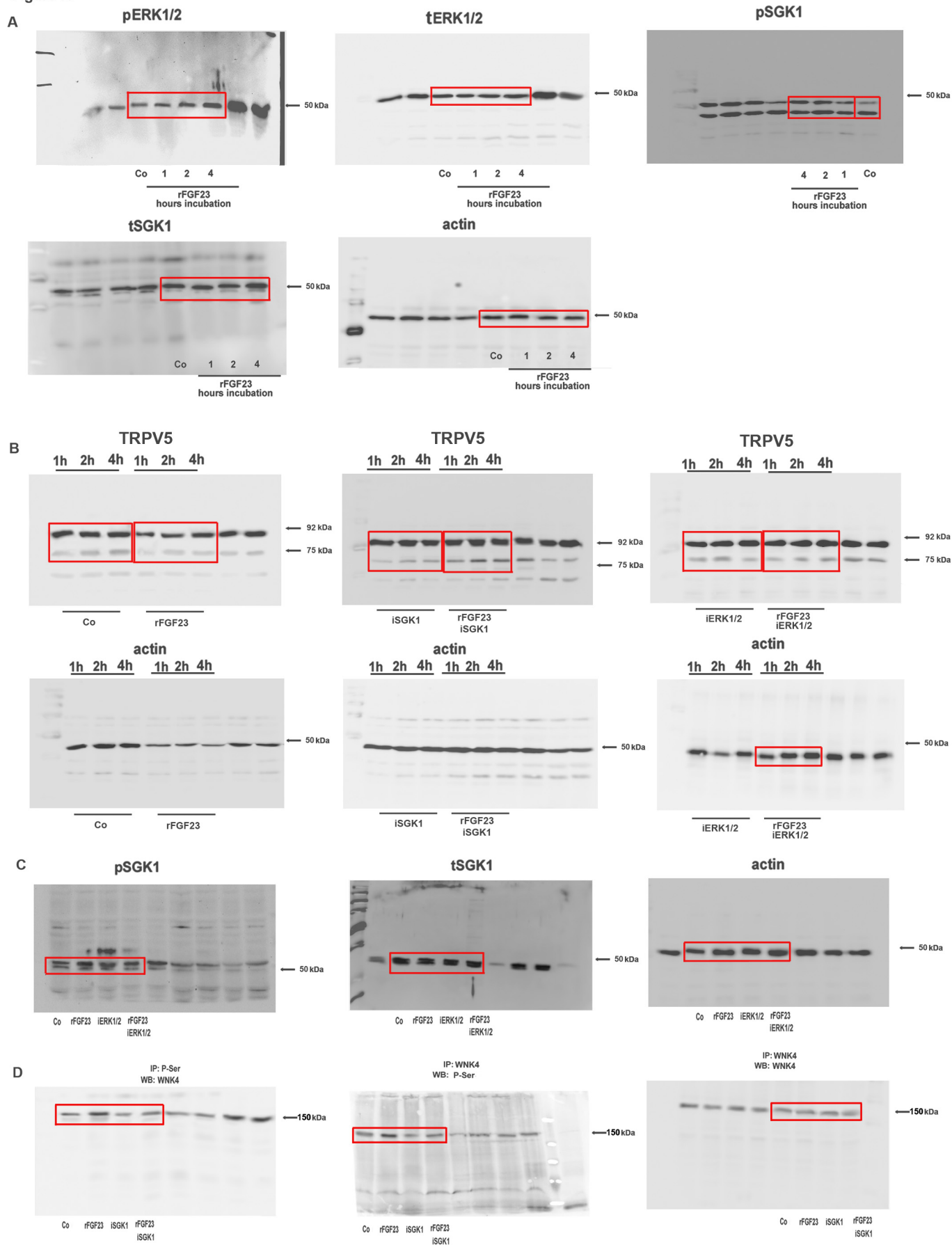

Supplement: Supplementary file 16 [file embj0033-0229-sd16.pdf]

Figure 8.

A

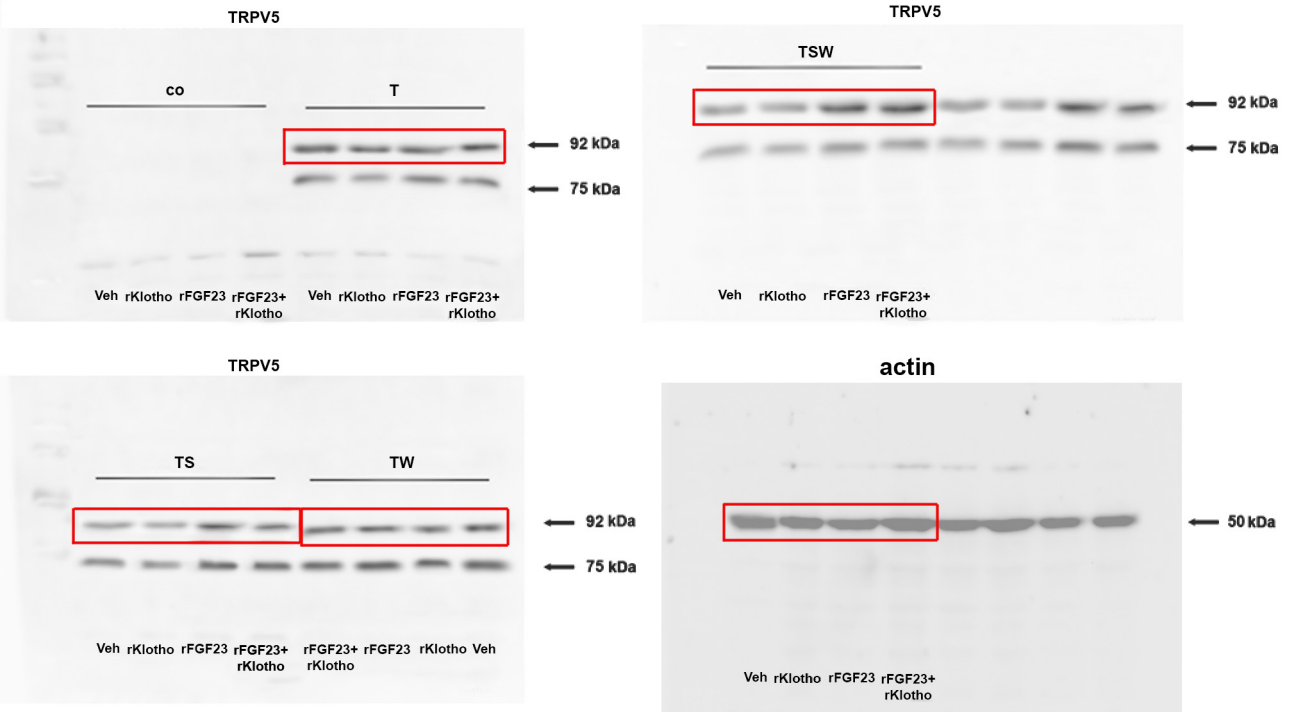

Supplement: Supplementary file 17 [file embj0033-0229-sd17.pdf]
